# Metabolomics of pulmonary exacerbations reveals the personalized nature of cystic fibrosis disease

**DOI:** 10.7717/peerj.2174

**Published:** 2016-08-11

**Authors:** Robert A. Quinn, Yan Wei Lim, Tytus D. Mak, Katrine Whiteson, Mike Furlan, Douglas Conrad, Forest Rohwer, Pieter Dorrestein

**Affiliations:** 1 Skaggs School of Pharmacy and Pharmaceutical Sciences, University of California, San Diego, CA, United States; 2 Department of Biology, San Diego State University, San Diego, CA, United States; 3 Mass Spectrometry Data Center, National Institute of Standards and Technology, Gaithersburg, MD, United States; 4 Department of Molecular Biology and Biochemistry, University of California, Irvine, CA, United States; 5 Department of Medicine, University of California, San Diego, CA, United States

**Keywords:** Personalized medicine, Cystic fibrosis, Metabolomics, Exacerbation, Mass spectrometry

## Abstract

**Background.** Cystic fibrosis (CF) is a genetic disease that results in chronic infections of the lungs. CF patients experience intermittent pulmonary exacerbations (CFPE) that are associated with poor clinical outcomes. CFPE involves an increase in disease symptoms requiring more aggressive therapy.

**Methods.** Longitudinal sputum samples were collected from 11 patients (*n* = 44 samples) to assess the effect of exacerbations on the sputum metabolome using liquid chromatography-tandem mass spectrometry (LC-MS/MS). The data was analyzed with MS/MS molecular networking and multivariate statistics.

**Results.** The individual patient source had a larger influence on the metabolome of sputum than the clinical state (exacerbation, treatment, post-treatment, or stable). Of the 4,369 metabolites detected, 12% were unique to CFPE samples; however, the only known metabolites significantly elevated at exacerbation across the dataset were platelet activating factor (PAF) and a related monacylglycerophosphocholine lipid. Due to the personalized nature of the sputum metabolome, a single patient was followed for 4.2 years (capturing four separate exacerbation events) as a case study for the detection of personalized biomarkers with metabolomics. PAF and related lipids were significantly elevated during CFPEs of this patient and ceramide was elevated during CFPE treatment. Correlating the abundance of bacterial 16S rRNA gene amplicons to metabolomics data from the same samples during a CFPE demonstrated that antibiotics were positively correlated to *Stenotrophomonas* and *Pseudomonas*, while ceramides and other lipids were correlated with *Streptococcus*, *Rothia*, and anaerobes.

**Conclusions.** This study identified PAF and other inflammatory lipids as potential biomarkers of CFPE, but overall, the metabolome of CF sputum was patient specific, supporting a personalized approach to molecular detection of CFPE onset.

## Introduction

Cystic fibrosis (CF) is a genetic disease caused by mutations in the cystic fibrosis transmembrane conductance regulator gene (CFTR). Mutations in CFTR result in airway mucus buildup and chronic airway infections. CF patients experience intermittent pulmonary exacerbations (CFPE), events that are poorly defined clinically, but known to lead to lung function decline and accelerated disease progression (Goss & Burns, 2007). A CFPE is characterized as an acute increase in symptom severity, such as dyspnea, cough, sputum production, chest pain, fevers, acute and chronic sinusitis, and occasionally hemoptysis (Dakin et al., 2001; Goss & Burns, 2007; Bilton et al., 2011; Stenbit & Flume, 2011). Exacerbations require an increased use of antibiotic, anti-inflammatory, and lung clearance therapies. These events decrease the quality of life of CF patients and a higher CFPE frequency is correlated with poor outcomes in one and three-year probability of death studies (De Boer et al., 2011; Aaron et al., 2014; Habib et al., 2015). CFPEs are challenging to predict (Rogers et al., 2011). Clinicians rely on symptoms common across patients or those that a single patient has previously experienced to provide some indication that a CFPE is occurring. Biomarkers that predict pulmonary exacerbations are needed to supplement existing clinical and physiological assessments (Rogers et al., 2011; Stenbit & Flume, 2011).

Omics approaches show great promise for the development of biomarkers as they generate large data sets containing thousands of variables that may be predictive (Ghosh & Poisson, 2009; Hu et al., 2011). With appropriate rigor and validation, metabolites are particularly good biomarkers, because they are easily detectable with analytical methods and can reflect imbalances in microbial or host metabolism that may explain disease pathology (Koulman et al., 2009; Li et al., 2013). For example, ceramide has been observed to accumulate in the epithelium of CFTR knockout mice (Teichgräber et al., 2008; Brodlie et al., 2010), with follow up studies linking CFTR to ceramide regulation (Bodas et al., 2011). The fermentation product 2,3-butanedione has been detected in the breath gas of CF patients; its source traced to streptococcal metabolism in the lung (Whiteson et al., 2014). Microbial growth signatures have also had value as predictive of exacerbation (Twomey et al., 2013; Carmody et al., 2013; Quinn et al., 2015a), although some studies find little change in microbial activity (Stressmann et al., 2011; Fodor et al., 2012). More comprehensive studies of metabolic and microbial changes through CFPE are needed, as well as an assessment of the molecular similarity across patients, to determine whether universal biomarkers can be identified or if a more personalized approach is required.

This study used LC-MS/MS based metabolomics on sputum from patients through exacerbation events. The sputum chemistry was more similar within individuals than within shared clinical states (exacerbation, stable, treatment, or post treatment). This supports a personalized approach to CFPE biomarker detection, and therefore, a case study of this personalized approach is presented on data collected from a single patient for over 4 years.

## Materials and Methods

Additional details are provided in the Supplemental Information 1.

### Sample collection

Sputum samples were collected at the adult cystic fibrosis center at the University of California at San Diego Medical Center as described in Lim et al. (2014). Procedures were approved under the UCSD Human Research Protections Program protocol #081500. Treating physicians determined which patients would be selected for the study, whether or not they were presenting with an exacerbation (principally based on the Fuchs criteria (Fuchs et al., 1994)), and the subsequent treatment regime. Samples were classified as ‘exacerbation,’ according to the clinicians diagnosis of the need for intravenous antibiotic administration, ‘treatment,’ as any sample collected during the 14 day treatment course, ‘post-treatment,’ as samples collected immediately after treatment course, and ‘stable,’ as samples collected during routine clinical visits that were not known at the time to be during an exacerbation. Sample collection involved an initial saline mouth rinse followed by expectoration of sputum into a sputum cup after inhalation of 7% hypertonic saline for 30 min and then syringe homogenized according to Lim et al. (2013). Two different sample sets were collected for assessment of metabolome similarity in different clinical states or between patients, one in 2012 and another in 2014 (Table S1). For a separate and more in depth longitudinal analysis, another sample set was collected from a single patient through 1,492 days (*n* = 37 sputa, Table S2). This sample set captured four separate exacerbation events, including samples that were collected daily for 14 days through treatment of the second exacerbation. Bacterial 16S rRNA gene profiles were previously published on sputum samples from this daily collection of the second CFPE event (Quinn et al., 2015a) and this data is also utilized in this study (see methods below and Table S2).

### Extraction and LC-MS/MS

The samples were thawed and then extracted using a sequential method of ethyl acetate solvation, followed by methanol solvation. A volume of 200 µl of sputum was first mixed with 200 µl of ethyl acetate, extracted for 1 h at room temperature and then briefly centrifuged. The supernatant was decanted and then evaporated in a centrifugal evaporator. The same volume of methanol was then added to the remaining sputum sample and incubated at room temperature for 1 h, and then briefly centrifuged. This supernatant was added to the dried pellet of the ethyl acetate extract and then evaporated in a centrifugal evaporator. All pellets were solubilized for 1 h in 100 µl of methanol prior to LC-MS/MS analysis. Mass spectrometry was performed using a Bruker Daltonics^®^ Maxis qTOF mass spectrometer equipped with a standard electrospray ionization source. A water/acetonitrile solvent separation gradient containing 0.1% formic acid was used from 98:2 to 2:98 water:acetonitrile for a total run time of 840 s. The mass spectrometer was operated in data dependent positive ion mode, automatically switching between full scan MS and MS/MS acquisitions.

### Metabolome generation and statistical analysis

For identification of the number of unique spectra in each clinical disease state MS/MS spectral alignments using molecular networking on GNPS (gnps.ucsd.edu) was performed on data from the 2012 and 2014 longitudinal collections. The number of unique spectra identified from molecular networking for each disease state was calculated and visualized using a Venn diagram.

Statistical analysis including multivariate comparisons and quantification of metabolite relative abundance was completed using MS^1^ traces. Molecular features detected in the mass spectrometer (MS^1^ level) were identified using the Bruker Daltonics^®^ DataAnalysis software and imported into the ProfileAnalyisis software version 2.1 (build 282, 64-bit) for metabolome generation on the entire data set. The 2012 and 2014 sputum datasets were processed and analyzed separately due to batch effects, and global similarity between patient and clinical state was only compared within each dataset. The metabolomes for dataset 1 and 2 were analyzed with a Bray–Curtis distance matrix separately using the *vegan* package in R (Oksanen et al., 2015). Each distance matrix was then projected with nMDS to visualize clinical state and patient metabolome similarity. The distance matrices were tested for similarity between the two classifiers using ANOSIM and PerMANOVA (adonis) from the *vegan* package in the R statistical software. The ANOSIM R statistic is a permutation test of the null hypothesis that within group variation is not greater than between group variation; an *R*-value above 0.4 is considered sufficient to reject the null hypothesis. The PerMANOVA test is a non-parametric multivariate analysis of variance using a pseudo-*F*-test. Here the larger the value of *F* the greater likelihood the null hypothesis of no differences between group variations is false and the *p*-value comes from permutations. We used the ANOSIM R and PerMANOVA F to test whether the metabolomic data classified more strongly by patient or clinical state.

To identify global biomarkers of CFPE, it was initially necessary to unify MS^1^ data from all cohorts and batches into a contiguous matrix containing spectral features from all samples. In order to do so a novel algorithm was used, which employs robust methodologies to overcome difficulties in identifying the same metabolite across multiple data sets (details in Supplemental Information 1). With this unified MS^1^ spectral data matrix, a supervised random forest using clinical state as the classifier produced a variable importance plot that identified differentially abundant features across clinical states. Ten features were identified as especially able to differentiate between the clinical states. These features were tested for significant differences using an ANOVA and Tukey’s test of significance (*p* < 0.05) and corrected for multiple comparisons. Biomarkers in the CF1 longitudinal dataset were identified first using a random forests classification based on disease state. The variable importance plot (VIP) of these random forests was then used to identify the 30 most differential features across the clinical states. These 30 features were then subjected to a one-way ANOVA and a Tukey’s test of significance with a Bonferroni correction (30 features compared for CF1 dataset, *p* < 0.00167).

### 16S rRNA gene sequencing and metabolome correlations

Bacterial relative abundance data at the level of genus that was previously generated on the same samples collected during the second exacerbation of the CF1 longitudinal collection (published in reference Quinn et al., 2015a) was used to compare correlations between the metabolome and microbiome variables. A correlation matrix was calculated such that the area under the curve for the abundance of each molecular feature was regressed against the normalized abundance of either *Pseudomonas*, *Rothia*, *Streptococcus*, *Stenotrophomonas* or total anaerobes (*Prevotella*, *Veillonella*, *Gamella* and *Oribacterium*). The Pearson correlation coefficient (*r*) of this regression was then used to build a network in the Cytoscape^®^ software.

### Metabolome annotation

LC-MS/MS files generated on the mass spectrometer were converted to the .mzXML format for dereplication and molecular networking. Spectra generated from the MS/MS acquisition were searched against the GNPS database (gnps.ucsd.edu) using the molecular networking algorithm (Watrous et al., 2012).

## Results

### Metabolome relationships between patient and clinical state

**Figure 1:**
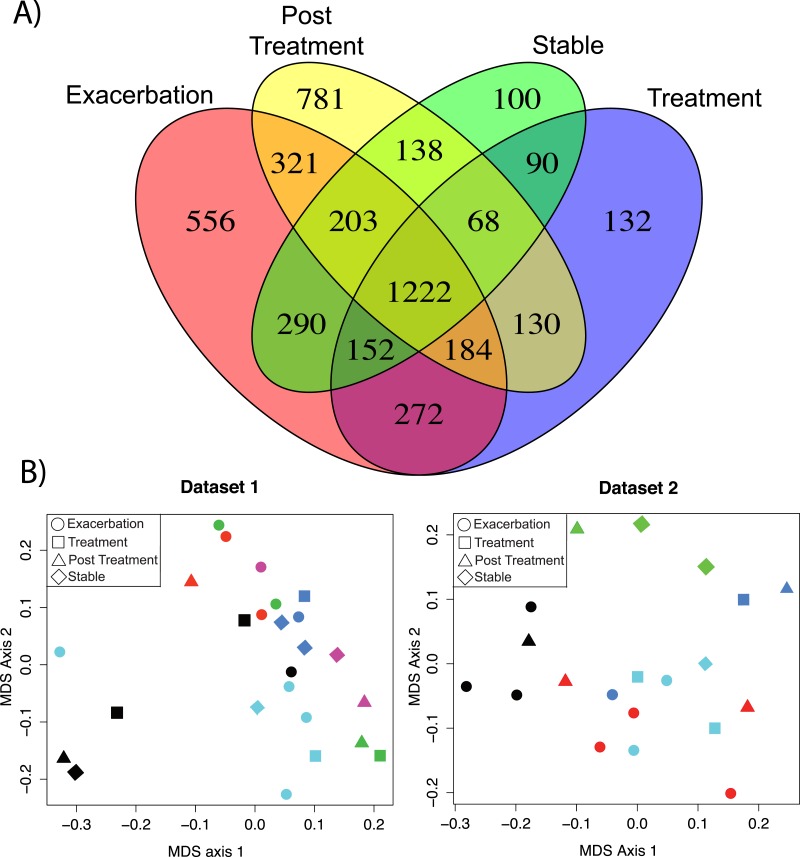
Comparison of metabolomics data across patients and disease state. (A) Venn diagram of membership of each unique MS/MS spectrum according to clinical state. (B) nMDS projection of a Bray–Curtis similarity matrix generated from metabolome data of dataset 1 and dataset 2, separately. The samples are colored by their patient of origin and shaped according to the clinical state at time of collection. The ANOSIM and PerMANOVA results of this similarity matrix are shown in Table 1.

Two sputum sample sets were collected for this study, one in 2011–2012 (*n* = 25 sputa, 6 patients) and another in 2014 (19 sputa, 5 patients, Table S1). Both data sets contain at least three samples per patient collected during one of 4 clinical states (Exacerbation ‘Ex,’ Treatment ‘Tr,’ Post Treatment ‘Pt,’ and Stable ‘St’). Technical variability of this method within a single run was assessed using multiple extractions of the same sample and on different days, revealing a highly reproducible method within a LC-MS/MS batch (Fig. S1, Supplemental Information 1 and 3). The data was analyzed with the molecular networking algorithm that identifies unique MS/MS spectra (a proxy for molecules) available through the Global Natural Product Social Molecular Networking database (GNPS, gnps.ucsd.edu, (Watrous et al., 2012)). A total of 4,639 unique MS/MS spectra were detected in the sample set. Exacerbation samples had 556 unique metabolites, Tr samples contained 132, Pt samples contained 781, St samples contained 100, and 1,222 metabolites were common across all clinical states (Fig. 1A).

Batch effects were detected between the sample sets by clustering of the metabolome data (Fig. S1). Due to these LC-MS/MS batch effects, the similarity of each metabolome could not be compared across the two datasets, only within each dataset. Therefore, a Bray–Curtis distance matrix was generated on the metabolite abundance matrix for each sample set separately and visualized with non-metric multidimensional scaling (nMDS) (Fig. 1B). The metabolomes were tested for similarity within the patient or clinical state classifiers using the analysis of similarity (ANOSIM) and permutation multivariate analysis of variance (PerMANOVA) (Fig. 1B and Table 1). The metabolomes clustered more strongly by patient than clinical state for both datasets (Fig. 1B). The ANOSIM statistic R (a measure of the difference of mean ranks between and within groups) was higher by patient classification than clinical state (for both datasets patient *R* > 0.4, Table 1). The PerMANOVA *F*-test verified this trend (for both datasets patient *F* ≥ 2.0, Table 1). This untargeted metabolomics analysis demonstrated that although there were a large number of unique molecules belonging to each clinical state, particularly exacerbation, the overall data did not reveal similarity in these clinical states across patients.

**Table 1:** Similarity of metabolomes between patient and clinical state classifiers. ANOSIM and perMANOVA results on Bray–Curtis dissimilarity of each abundance matrix for the two longitudinal datasets analyzed.

	Patient		Clinical state	
Statistic	ANOSIM R (p)	perMANOVA F (p)	ANOSIM R (p)	perMANOVA F (p)
Dataset 1	0.485 (*p* = 0.001)	3.07 (*p* = 0.001)	0.076 (*p* = 0.772)	0.759 (*p* = 0.886)
Dataset 2	0.553 (*p* = 0.001)	2.00 (*p* = 0.001)	0.196 (*p* = 0.060)	1.269 (*p* = 0.074)

### Global biomarkers of CFPE

**Figure 2:**
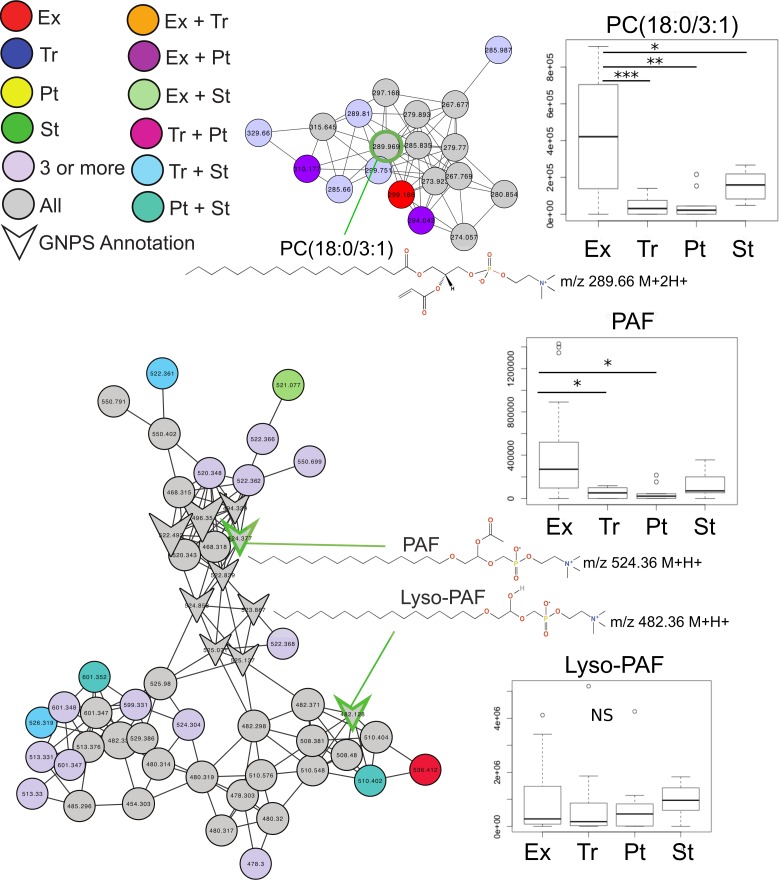
Molecular network of exacerbation biomarkers. Molecular network clusters containing two significant biomarkers of exacerbation identified in the CF sputum metabolome data. Nodes are colored according to which clinical state(s) they were detected in and shaped according to whether they were automatically annotated through GNPS. Each cluster represents a molecular family of molecules related to the biomarker metabolite identified using a supervised random forests with clinical state as a classifier. Nodes of interest are highlighted by a green outline. The chemical structure of the biomarker and important molecular relatives are also shown. Boxplots of the area under the curve abundance of each biomarker is shown in each clinical state with significance according to the Tukey’s test (^∗^, *p* < 0.05; ^∗∗^, *p* < 0.01; ^∗∗∗^, *p* < 0.001). Exact locations of the double bond in the PC molecules are unknown.

Common features across the two sample sets were identified using an advanced retention time alignment and feature detection method (Fig. S1, Supplemental Information 1 and 3). Although the majority of metabolites unique to exacerbation were patient specific, some were significantly elevated across patients. These were identified using a supervised random forests (Liaw & Wiener, 2002) approach with the samples classified by clinical state on the merged abundance matrix between the two sample sets. Data from both sets were merged using a novel algorithm expressly designed to overcome inter-batch *m*∕*z* and retention time variations in order to maximize the number of metabolites common to both sets (see Supplemental Information 1). The variable importance plot revealed eight metabolites had a mean decrease in accuracy of classification above 4, indicating they were highly enriched in a particular disease state, the remaining metabolites contributed less to the classification (Fig. S2). Of these eight metabolites, MS/MS spectral matching and GNPS library searching allowed putative annotations for two. Platelet activating factor (*m*∕*z*524.36 (*M* + *H*^+^)) was significantly more abundant in Ex than Tr and Pt states, but not during St (Tukey’s test of ANOVA, Ex–Tr *p* = 0.021, Ex–Pt *p* = 0.022, Fig. 2). The diacylglycerophosphocholine lipid PC(18:0/3:1) was significantly more abundant in Ex than all other clinical states (Ex–Tr *p* = 0.0062, Ex–Pt *p* = 0.003, Ex–St *p* = 0.038) (Fig. 2). Lyso-PAF, a related metabolite, was not significantly different between clinical states.

### Personalized biomarkers of CFPE

**Figure 3:**
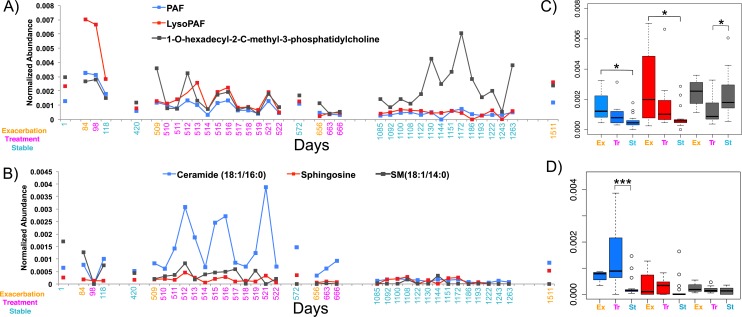
The normalized abundance of specific known metabolites identified through GNPS in CF1 over 1,492 days. Date of sample collection is shown on the *x*-axis and the clinical state of the sample is colored according to the legend. (A) The Lyso-phospholipids PAF, Lyso-PAF and 1-O-hexadecyl-2-C-methyl-3-phosphatidylcholine. (B) The sphingolipids ceramide (18:1/16:0), sphingosine and sphingomyelin (18:1/14:0). Boxplots of the distribution of the abundances of the lysophospholipids (C) and sphingolipids (D) in the different disease states, boxes are colored according to the legend in the panel of the adjacent line graph.

In light of the individuality observed between CF sputum metabolomes, the remainder of this study focused on a single patient with longitudinal sampling over a four-year time period, which captured four separate exacerbation events (CF1 dataset, Table S2, *n* = 37 sputa). Known spectra detected in the CF1 longitudinal dataset were identified through the GNPS molecular networking workflow and library searching and their abundances tracked through time (Figs. 3A and 3B). The abundance of PAF, Lyso-PAF and a monoacylglycerophosphocholine lipid Lyso-PC (1-O-hexadecyl-2-C-methyl-3-phosphatidylcholine) were monitored through the data set and revealed fluctuations in the abundance of these metabolites through time (Fig. 3A). PAF and Lyso-PAF were elevated during Ex and Tr and the Lyso-PC was elevated during a 6-month period of stability (Fig. 3A). A Tukey’s test of a one-way ANOVA for each molecule across the clinical states Ex, Tr and St (no Pt samples were available from this patient) showed that PAF and Lyso-PAF were significantly more abundant during Ex than during St (PAF *p* = 0.035, Lyso-PAF *p* = 0.039), but not Tr (Fig. 3C). PAF and Lyso-PAF abundance was highly correlated through the sampling period (Pearson’s *r* = 0.96, *p* < 0.0001), but not Lyso-PC (Pearson’s *r* = 0.25, *p* = 0.13). Sphingolipids were also identified in this patient through GNPS library searching including ceramide, sphingomyelin and sphingosine. Ceramide was especially elevated during treatment of an exacerbation (Fig. 3B). The Tukey’s test revealed that this molecule was significantly more abundant during Tr compared to St (*p* = 0.0008, Fig. 3D). Ceramide and sphingosine were significantly correlated in their abundances (Pearson’s *r* = 0.53, *p* = 0.0007), but sphingomyelin and ceramide were not (Pearson’s *r* = 0.27, *p* = 0.10). These known molecules may represent specific biomarkers of CF1 associated with inflammation during the onset of an exacerbation and through its treatment.

Other molecules that were not annotated through GNPS were also statistically significant biomarkers of CFPE for this patient. Thirty of these metabolites were identified using a random forests based classification of the entire CF1 longitudinal data set based on clinical state (Fig. S3). Molecular features with parent masses *m*∕*z*441.317, *m*∕*z*304.280, *m*∕*z*508.331, and *m*∕*z*551.354 were all significantly elevated during Ex and Tr, compared to St, after a Bonferroni correction for a Tukey’s test of a one-way ANOVA (*p* < 0.00167, 30 comparisons, Figs. S4 and S5). Metabolites *m*∕*z*508.331 and *m*∕*z*551.354 both contained a phosphocholine fragment in their MS/MS spectra (Fig. S4), indicating that they are putatively a monoacylglycerophosphocholine and a glycerophospholipid of unknown structure, respectively. Although these metabolites remain unidentified these molecules are especially strong biomarkers of exacerbation in CF1.

### Xenobiotic dynamics in CF1

**Figure 4:**
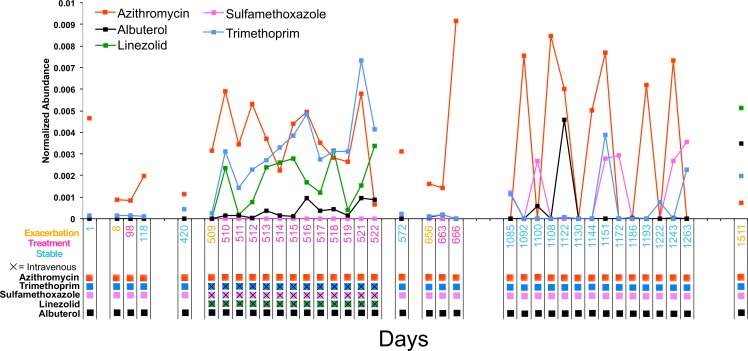
Longitudinal dynamics of antibiotics in a single patient over 4 years. Graph of the normalized abundance of xenobiotics in the CF1 longitudinal dataset. Date of sample collection is shown on the *x*-axis and the clinical state of the sample is colored according to the legend. Clinical administration of particular xenobiotics according to clinical treatment history is shown below the *x*-axis and colored according to the figure legend.

The antibiotics azithromycin, trimethoprim, sulfamethoxazole, and linezolid, and the bronchodilator albuterol were detected in the longitudinal sampling data of CF1. There was varying abundance of these antibiotics that fluctuated through time (Fig. 4). Azithromycin, trimethoprim, sulfamethoxazole, and albuterol were administered consistently as ‘suppressive’ therapy to this patient throughout the sampling period. During the second exacerbation event, trimethoprim, sulfamethoxazole and linezolid, were administered intravenously. The abundance of azithromycin was high in sputum through most of the sampling period, but fluctuated during suppressive administration, occasionally out of the detectable range. As expected, the abundance of trimethroprim and linezolid increased during intravenous therapy, but sulfamethoxazole remained at a very low abundance. Albuterol also increased in abundance during the second exacerbation (this metabolite was not part of the intravenous treatment regime, but was administered), while being relatively low during chronic oral therapy.

### Microbial and metabolome correlations

**Figure 5:**
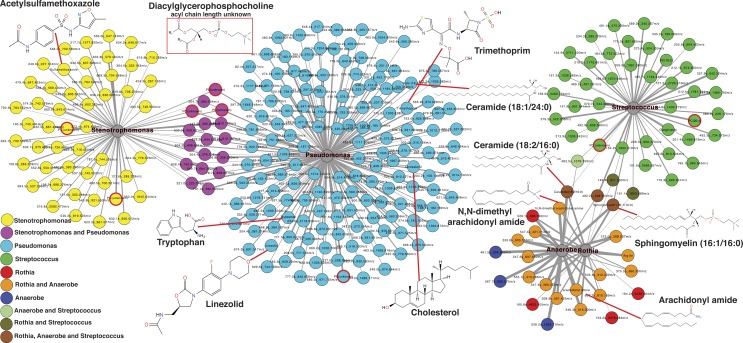
Correlation between microbes and molecules during a single exacerbation. Microbial and molecule correlation network built using Pearson’s *r* regression score between the normalized abundance of molecules and the normalized abundance of bacterial genera from 16S rDNA sampling in the same samples. The value of *r* is scaled to the thickness of the edge connections and the nodes are colored by their microbial associations. Annotated metabolite putative structures are highlighted.

The second exacerbation event during the CF1 longitudinal sampling also had bacterial 16S rDNA amplicon sequencing data previously published in a separate manuscript on the same sputum samples (Quinn et al., 2015a). Therefore, abundances of *Pseudomonas*, *Stenotrophomonas*, *Streptococcus*, *Rothia* and anaerobes (*Veillonella, Prevotella, Gemella* and *Oribacterium*) from the published data were regressed against the metabolite abundances in the same CFPE1 samples (*n* = 16). Statistically significant Pearson correlations (*p* < 0.05) were visualized by networking analysis using Pearson’s *r* as the edge values, such that molecules that were positively correlated with a particular bacterial genus would be connected in the network (Fig. 5). The network topology revealed that there were 168 molecular features significantly correlated with *Pseudomonas* abundance, 66 with *Stenotrophomonas*, 46 with *Streptococcus*, 23 with *Rothia*, and 22 with anaerobes. The network topology showed two groups of molecules, those that correlated with either *Stenotrophomonas* or *Pseudomonas* and those that correlated with *Streptococcus*, *Rothia*, and anaerobes (Fig. 5). A number of molecules were correlated to more than one bacterium, but there were no correlations between these two groups of bacteria. This indicated that there were two microbial and molecular communities in this patient during this exacerbation, one associated with *Stenotrophomonas* and *Pseudomonas* and one with Gram-positives and anaerobes. These two communities have been previously identified as the *Climax* and *Attack* communities, respectively, from a parallel study of these samples (Quinn et al., 2015a).

Automatic metabolite annotation through GNPS led to putative identification of 14 of the 303 molecules in the correlation network. Ceramide (18:2/16:0) and sphingomyelin (16:1/16:0) were correlated with *Streptococcus*, *Rothia* and anaerobes. N,N-dimethyl arachidonly amide and arachidonyl amide were correlated to *Rothia* and anaerobes, but not *Streptococcus*. Two diacylglycerophosphocholines were correlated with presence of *Streptococcus*. A number of antibiotics were correlated with *Stenotrophomonas* and *Pseudomonas* including trimethoprim, linezolid and acetylsulfamethoxazole, all three were administered intravenously as treatment for this exacerbation. Cholesterol, tryptophan, another diacylglycerophosphocholine, and ceramide (18:1/24:0) were correlated specifically with *Pseudomonas* relative abundance.

## Discussion

CF exacerbations negatively impact patient quality of life and accelerate lung function decline (De Boer et al., 2011). Antibiotic treatment is most often used to reduce the flare of acute symptoms (Horsley et al., 2013), but patients often have lingering effects and 25% of those treated do not return to their previous level of lung function (Sanders et al., 2010; Sanders et al., 2011). There is a need to identify the onset of these events before they develop to facilitate more effective treatment and minimize permanent lung remodeling. Biomarkers that could be longitudinally monitored to provide predictive value of the probability of an imminent CFPE would greatly aid clinicians. However, significant challenges exist in identifying ubiquitous biomarkers for CF due to the heterogeneity between patients in disease presentation (Collaco et al., 2010; Horsley & Siddiqui, 2015) and microbial infections (Carmody et al., 2013; Lim et al., 2013).

This study assessed the metabolomic changes that occurred through exacerbations in CF patients. A collective assessment of the similarity in the longitudinal sputum metabolomes from 11 different patients demonstrated that there was not a universal signature of exacerbation. Instead, the chemistry of sputum was more similar within individuals longitudinally than between different patients at the same clinical state of disease. Cystic fibrosis manifests in heterogeneous phenotypes, but the reasons for this are not clear (Collaco et al., 2010; Horsley & Siddiqui, 2015). At least 1,700 mutations in CFTR have been described and the clinical relevance of most of them is unknown (Bareil et al., 2010). Moreover, there are five described classes of CFTR mutations that disseminate into CF, but even within the same genotype class, the presentation of disease can be quite variable (Rowntree & Harris, 2003). The data in this study supports the heterogeneous nature of CF, where patients were found to have different chemical populations within their lung. It is well known that microbial profiles of patients are often unique (Carmody et al., 2015), this study indicates that the collective chemistry of sputum is also very specific to the individual.

A total of 556 molecules were unique to the Ex clinical state that could represent potential CFPE biomarkers for future screening (Fig. 1), however, the supervised random forests classified by clinical state demonstrated that few of these were significantly elevated across the patient cohort (Fig. S1). Two of the universally elevated molecules were PAF and a related molecule PC (18:0/3:1). The elevation of these two metabolites may indicate lipid remodeling during exacerbation. PAF is a particularly intriguing biomarker of CFPE, as it is a known inflammatory signaling lipid (Camussi, Tetta & Baglioni, 1990; Welch et al., 2009; Yost, Weyrich & Zimmerman, 2010) and has been reported to be elevated in exacerbations of other inflammatory lung diseases, such as asthma (Schauer et al., 1992; Kasperska-Zajac, Brzoza & Rogala, 2008). PAF and Lyso-PAF have opposing effects on neutrophil activation, the former activating neutrophils, and the latter deactivating them (Welch et al., 2009). Monitoring the flux through PAF and other related lipids might be an effective means of identifying increased neutrophilic inflammation and the potential onset of exacerbations.

**Figure 6:**
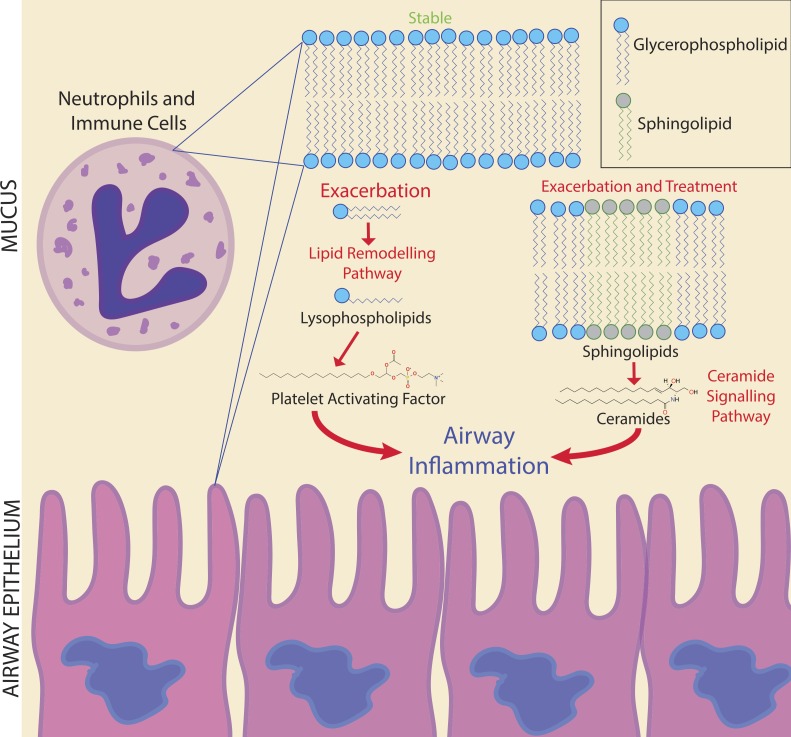
Potential biomarker pathways of CFPE onset identified in this study. Potential biomarker pathways of CFPE onset identified in this study. Lipid remodeling through PAF and ceramide may indicate inflammatory responses occurring during the onset and treatment of a CFPE. Membranes of neutrophils and epithelial cells contain these lipids and are the likely source of these signatures.

Due to the personalized nature of sputum chemistry, a single patient was monitored with metabolomics for 4.2 years, as a case study for personalized medicine biomarker development. This dataset captured four unique exacerbation events. Known molecules identified through GNPS spectral matching (including PAF-like lipids, sphingolipids and specific drugs) were monitored for changes in their abundance through time with attention to different disease classifications of each sample. PAF and Lyso-PAF had highly correlated abundances. This may indicate that PAF is being synthesized through phospholipid breakdown, first through the production of Lyso-PAF by phospholipase A2, then acetylation by Lyso-PAF acetyltransferase. This is the classic remodeling pathway of PAF synthesis and is associated with pathology and inflammation (Uemura, Lee & Snyder, 1991) (Fig. 6). PAF production may be another host physiological response to exacerbation and an important signature of an increased inflammatory load. Sphingolipids were also followed through the CF1 longitudinal data set. Ceramide, another potent inflammatory signaling lipid (Nixon, 2009), was especially abundant during treatment of an exacerbation. This too may represent a heightened inflammatory response, or influx of ceramide containing inflammatory cells in response to CFPE (Fig. 6). Together, PAF and ceramide are potentially strong candidates as CFPE biomarkers across patients or within certain individuals. The pathways of their production in the context of the CF lung microenvironment are illustrated in Fig. 6. This figure depicts the potential biomarkers detected in this study and their likely source from the membranes of both epithelial and recruited inflammatory cells.

Changes in sputum at CFPE onset have been observed in other studies, including a breath gas study and a sputum study using metabolomics (Twomey et al., 2013; Whiteson et al., 2014). Here we demonstrate changes in the lipid pool within a single patient through time. The fluctuations in sphingolipids and overall elevation of ceramide during CFPE treatment indicate that sphingolipid signaling may be dynamic through an exacerbation. Because cells deficient in CFTR have been shown to accumulate ceramide in the epithelium (Teichgräber et al., 2008; Bodas et al., 2011; Brodlie et al., 2010; Ziobro et al., 2013), CFTR mutations may be responsible for the buildup of ceramide in sputum (Quinn et al., 2015b), which is then observed as large fluctuations in ceramide signaling in response to inflammation induced at the onset of exacerbation. Although this study represents a relatively small sample size, these results support further research into the role of ceramide and ceramide induced signaling in CF as a potential therapeutic target (Brodlie et al., 2010; Becker et al., 2011; Ziobro et al., 2013) and biomarker of exacerbation. Larger population studies will be needed to determine if PAF and ceramide could be universally useful biomarkers or if they may be more patient specific.

A number of unknown metabolites were significantly elevated at exacerbation for CF1. While these were not automatically annotated molecules through GNPS, in the context of personalized biomarker detection for CFPE, metabolite signatures do not need to be known spectra to be to clinically relevant. If they are statistically robust and provide a consistent LC-MS/MS signature, they may be just as effective in identifying CFPE onset as known metabolites. Annotation of the thousands of spectra generated in a mass spectrometry experiment is a significant challenge in the metabolomics field (Da Silva, Dorrestein & Quinn, 2015). As databases and algorithms improve, the ability to annotate the most statistically robust metabolites will be more attainable for any clinical biomarker. This will greatly improve the application of mass spectrometry to the clinical environment, particularly for personalized and precision medicine approaches.

Drug metabolism and penetration to the target site is an important aspect of treatment not easily assessed during routine clinical regimes. Azithromycin, constitutively prescribed to CF1 throughout the four years of sample collection, had dynamic fluctuations in this patient through the four years of study. Whether this is due to poor treatment adherence or time since actual patient ingestion cannot be known from the data collected in this study, but the fluctuating abundance of this xenobiotic may have a specific effect on the metabolome and microbiome of the lung. Intravenous administration of an antibiotic results in a more sustained presence in CF sputum, as is shown for trimethoprim, sulfamethoxazole and linezolid. This indicates that this form of therapy does result in the presence of high amounts of these molecules in lung mucus throughout the two-week treatment course.

Bacterial 16S rRNA microbiome profiles of sputum from CF1 were also generated on the same samples collected during the intravenous therapy for the second exacerbation (Quinn et al., 2015a). This allowed for a unique opportunity to monitor the changes in sputum microbiome along with metabolome using correlation. The network topology of the correlations between microbial and metabolite relative abundance identified the contrasting microbial communities previously proposed to exist in CF lungs (Conrad et al., 2012). Many molecules were positively correlated to *Pseudomonas* and *Stenotrophomonas* abundance, as well as *Streptococcus*, *Rothia* and anaerobes. However, there were no cross correlations between these groups, indicating that separate microbial communities may have separate chemical communities associated with them. Trimethoprim and linezolid were correlated with the *Pseudomonas* and *Stenotrophomonas*, indicating the inherent resistance of these microbes to the intravenous antibiotic treatment (Zhang, Li & Poole, 2000; Betriu, 2001; Lister, Wolter & Hanson, 2009). Ceramide and arachidonyl amides were associated with the Gram-positive and anaerobic community. Combining microbiome sequencing with the ability of LC-MS/MS and molecular networking through GNPS to automatically identify both host, microbial and xenobiotic metabolites represents a powerful clinical tool to monitor the effect of antibiotic therapy on lung chemistry and microbiology.

## Conclusions

The chemistry of sputum samples compared in this study was more similar within patients through time than across patients with the same clinical state. Although there was not a universal signature of exacerbation, personalized approaches to biomarker development show promise, as a large number of metabolites were unique to this clinical state. Monitoring an individual through multiple exacerbations could provide statistically robust molecular biomarkers with future predictive value. Here, PAF and ceramide are potentially useful biomarkers as inflammatory lipids indicating the potential onset of a CFPE. Monitoring lipid remodeling through the classical PAF and ceramide pathways may provide information about inflammatory processes occurring prior to and during a CFPE event (Fig. 6). Because mass spectrometry-based metabolomics can be completed in clinically relevant time frames, application of these methods to screen for fluctuations in the abundance of metabolites associated with clinically relevant phenotypes is a powerful approach to personalized medicine. The metabolomics analysis methods applied to this single patient represents a proof-of-principle that personalized metabolomics can be used to study chemical dynamics during exacerbation. Further research concerning the role of CFTR in ceramide induced hyperinflammation associated with exacerbations may lead to novel treatment approaches to reduce the damage caused by a CFPE.

##  Supplemental Information

10.7717/peerj.2174/supp-1 Figure S1 Mean decrease in accuracy rank of metabolites identified in the variable importance plot of the merged multi-patient longitudinal data set Random forests was run with each sputum sample classified by the clinical state at the time of its collection. Fig. S2. Variable importance plot of the CF1 longitudinal data set random forests classified by clinical state. The *m*∕*z* value of each metabolite is shown on the *y*-axis. Fig. S3. Boxplots of the normalized abundance of unknown biomarkers detected in the CF1 longitudinal dataset. Figure S4. Extracted ion chromatogram and MS/MS spectra of unknown biomarkers identified in CF1 longitudinal dataset.

10.7717/peerj.2174/supp-2 Figure S2 Mean decrease in accuracy rank of metabolites identified in the variable importance plot of the merged multi-patient longitudinal data set Mean decrease in accuracy rank of metabolites identified in the variable importance plot of the merged multi-patient longitudinal data set. Random forests was run with each sputum sample classified by the clinical state at the time of its collection.

10.7717/peerj.2174/supp-3 Figure S3 Variable importance plot of the CF1 longitudinal data set random forests classified by clinical state Variable importance plot of the CF1 longitudinal data set random forests classified by clinical state. The *m*∕*z* value of each metabolite is shown on the y-axis.

10.7717/peerj.2174/supp-4 Figure S4 Boxplots of the normalized abundance of unknown biomarkers detected in the CF1 longitudinal dataset

10.7717/peerj.2174/supp-5 Figure S5 Extracted ion chromatogram and MS/MS spectra of unknown biomarkers identified in CF1 longitudinal dataset

10.7717/peerj.2174/supp-6 Table S1 Supplemental tables Patients and disease states for longitudinal sputum samples in this study. Table S2. Longitudinal sputum samples collected from patient CF1 in this study.

10.7717/peerj.2174/supp-7 Supplemental Information 1 Supplementary Methods

10.7717/peerj.2174/supp-8 Supplemental Information 2 Manuscript where 16S rRNA sequences were originally published

10.7717/peerj.2174/supp-9 Supplemental Information 3 Supplementary Results Supplementary results describing technical variability and batch effects

## Additional Information and Declarations

### Data Availability

The following information was supplied regarding data availability: GNPS (gnps.ucsd.edu): MSV000079104, MSV000079443, MSV000079444.
