# Supplementary figures and images for: Metabolomics of pulmonary exacerbations reveals the personalized nature of cystic fibrosis disease
Source: PeerJ. 2016 Aug 11;4:e2174. doi: 10.7717/peerj.2174 (PMC4991883; doi:10.7717/peerj.2174)

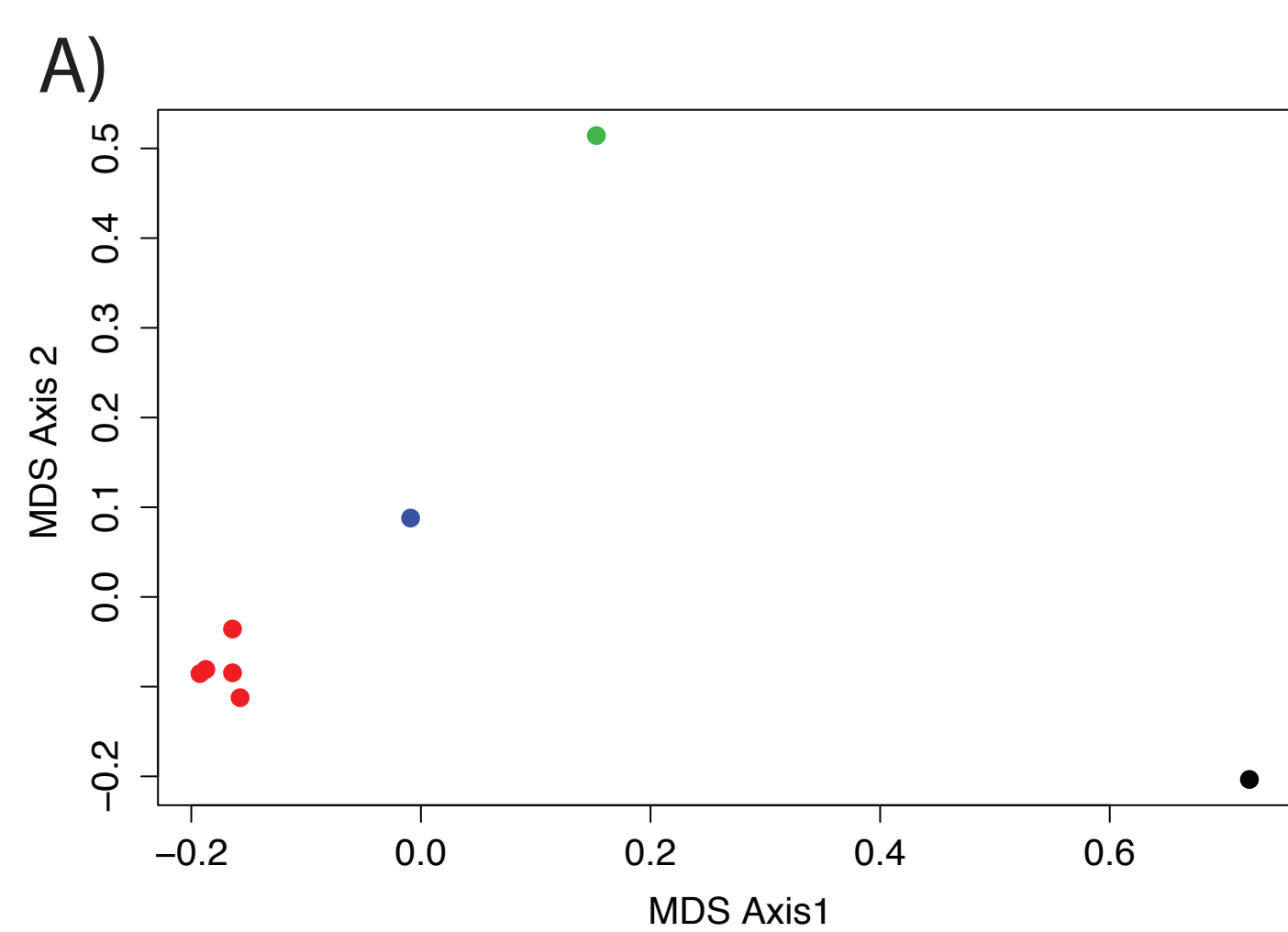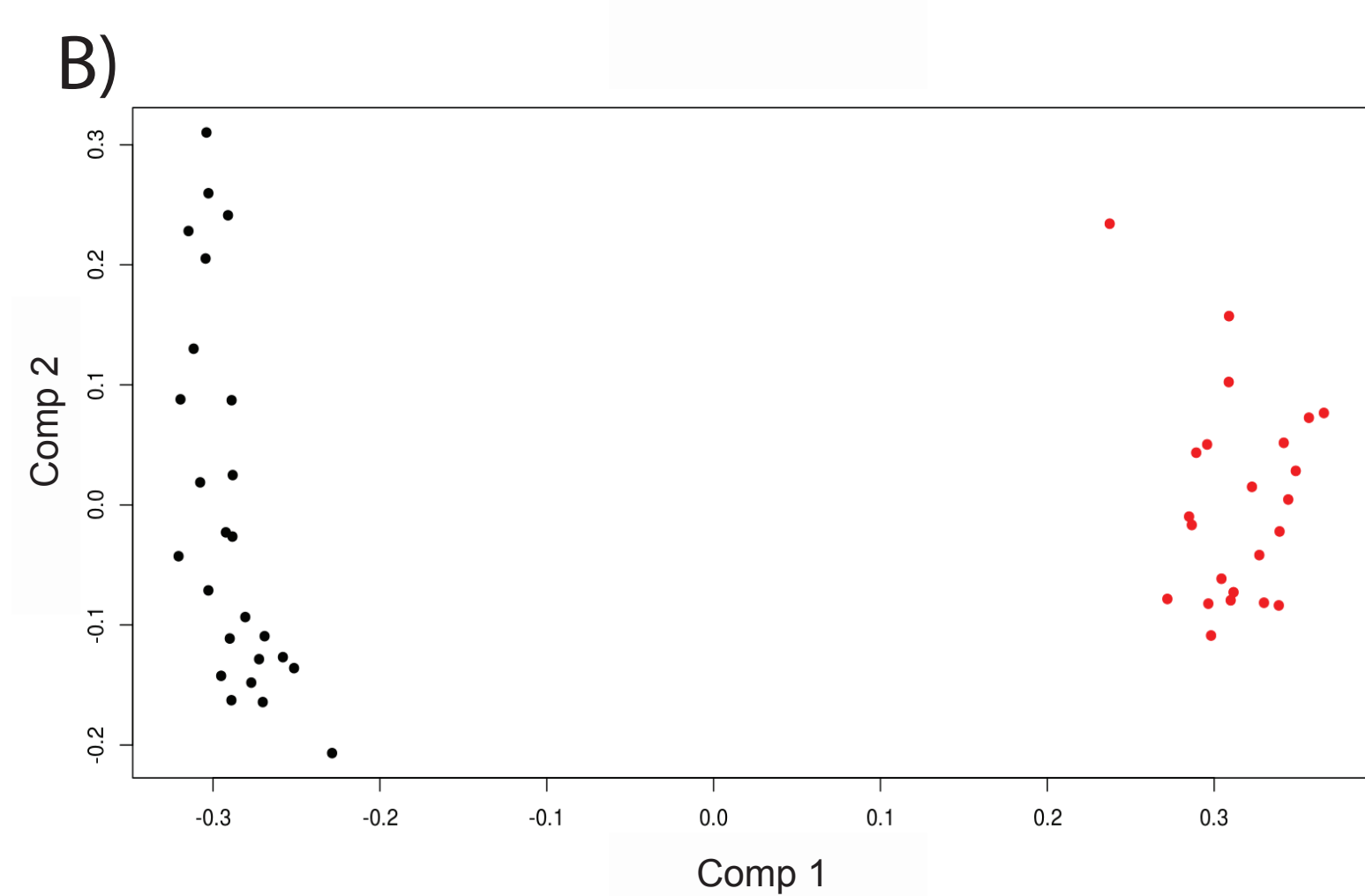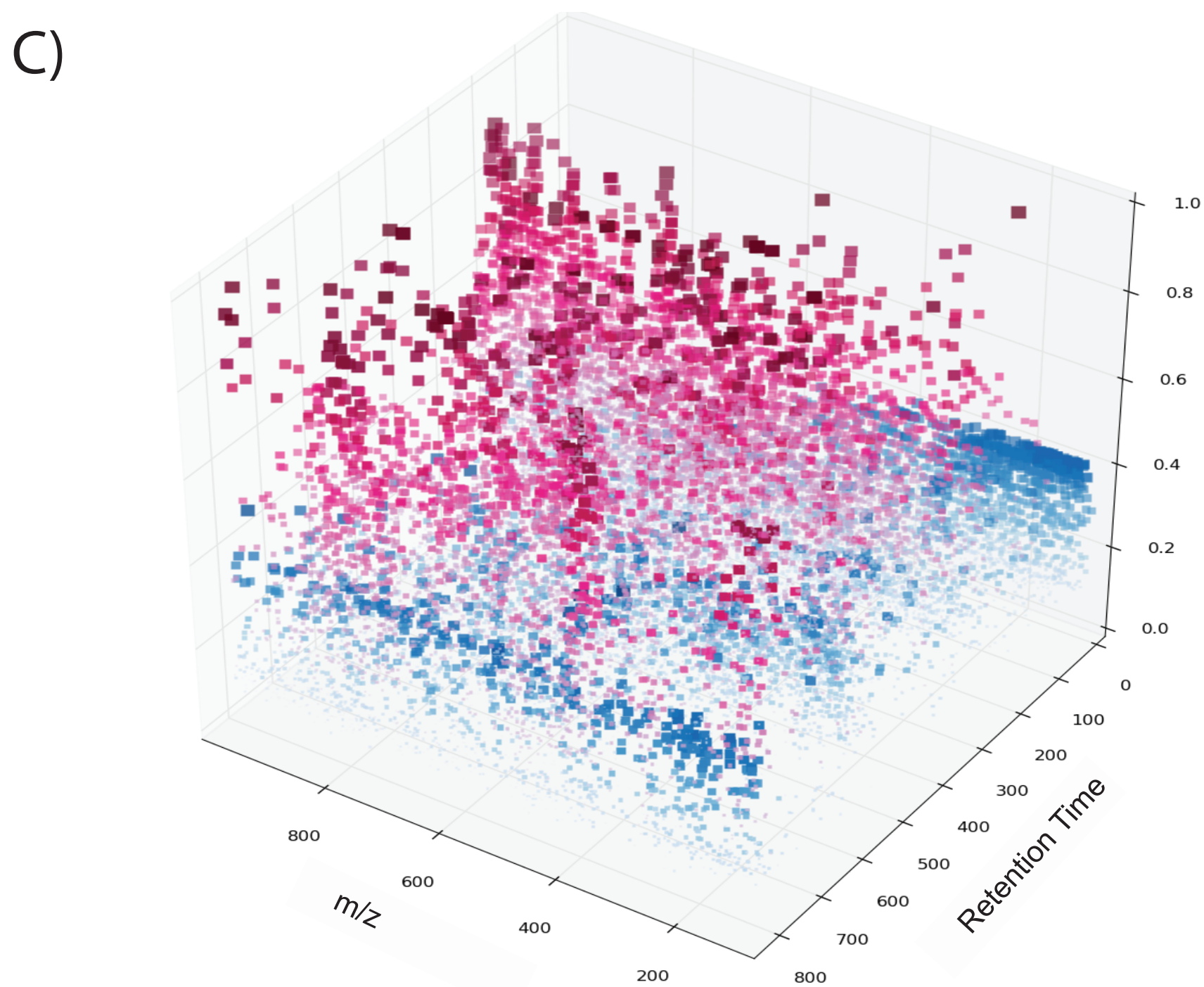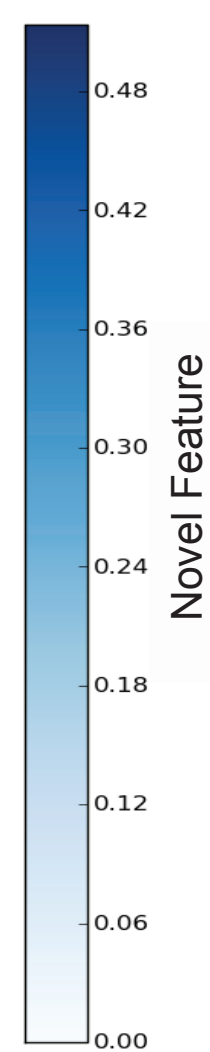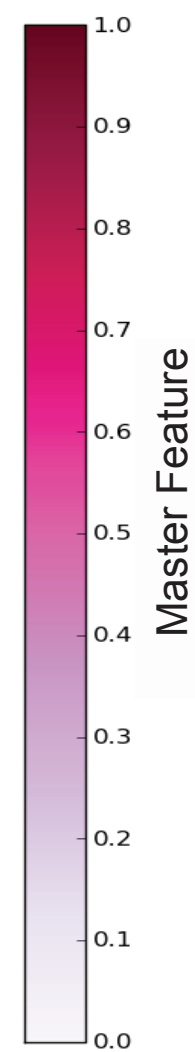

Supplement: Figure S1 — Random forests was run with each sputum sample classified by the clinical state at the time of its collection. Fig. S2. Variable importance plot of the CF1 longitudinal data set random forests classified by clinical state. The m∕z value of each metabolite is shown on the y-axis. Fig. S3. Boxplots of the normalized abundance of unknown biomarkers detected in the CF1 longitudinal dataset. Figure S4. Extracted ion chromatogram and MS/MS spectra of unknown biomarkers identified in CF1 longitudinal dataset. [file peerj-04-2174-s001.pdf]

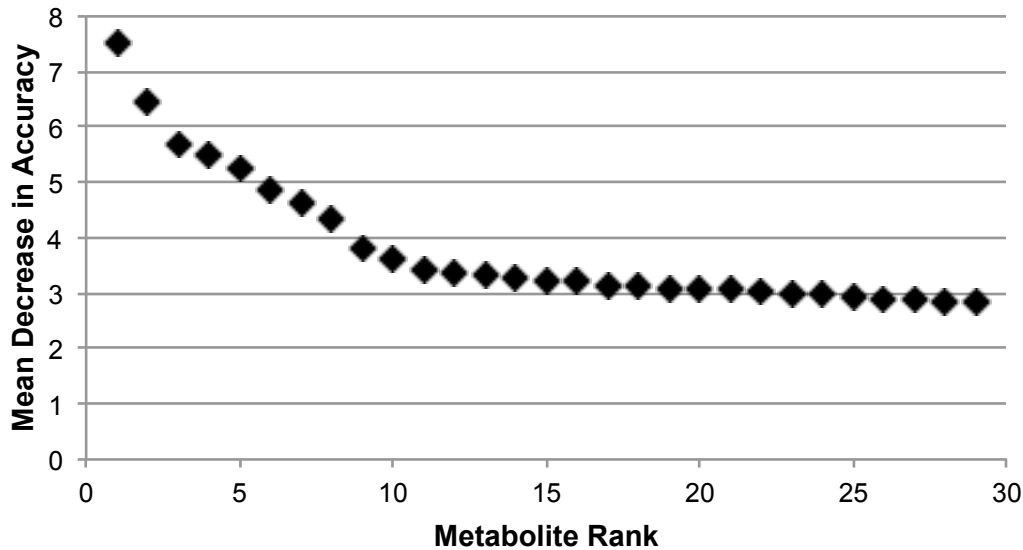

Supplement: Figure S2 — Mean decrease in accuracy rank of metabolites identified in the variable importance plot of the merged multi-patient longitudinal data set. Random forests was run with each sputum sample classified by the clinical state at the time of its collection. [file peerj-04-2174-s002.pdf]

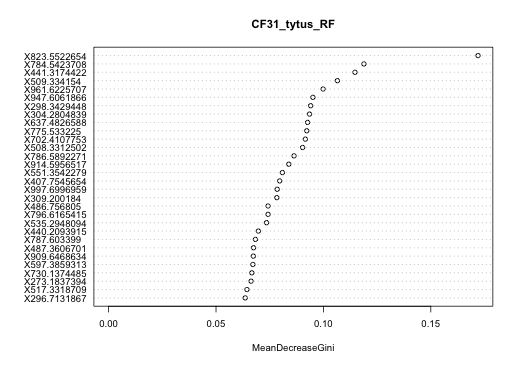

Supplement: Figure S3 — Variable importance plot of the CF1 longitudinal data set random forests classified by clinical state. The m∕z value of each metabolite is shown on the y-axis. [file peerj-04-2174-s003.png]

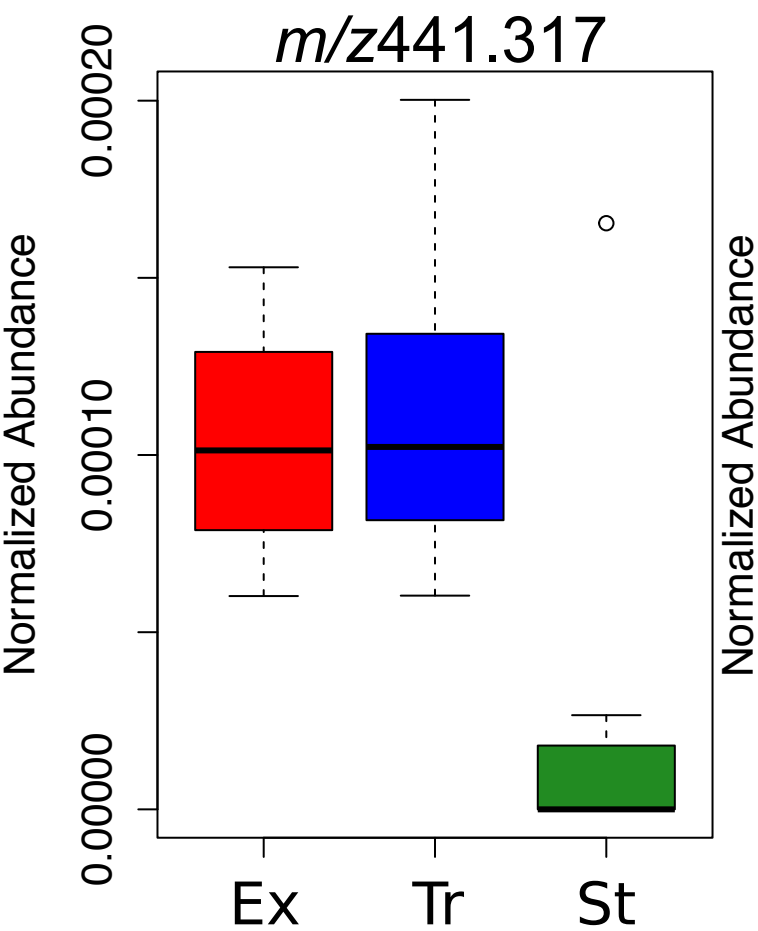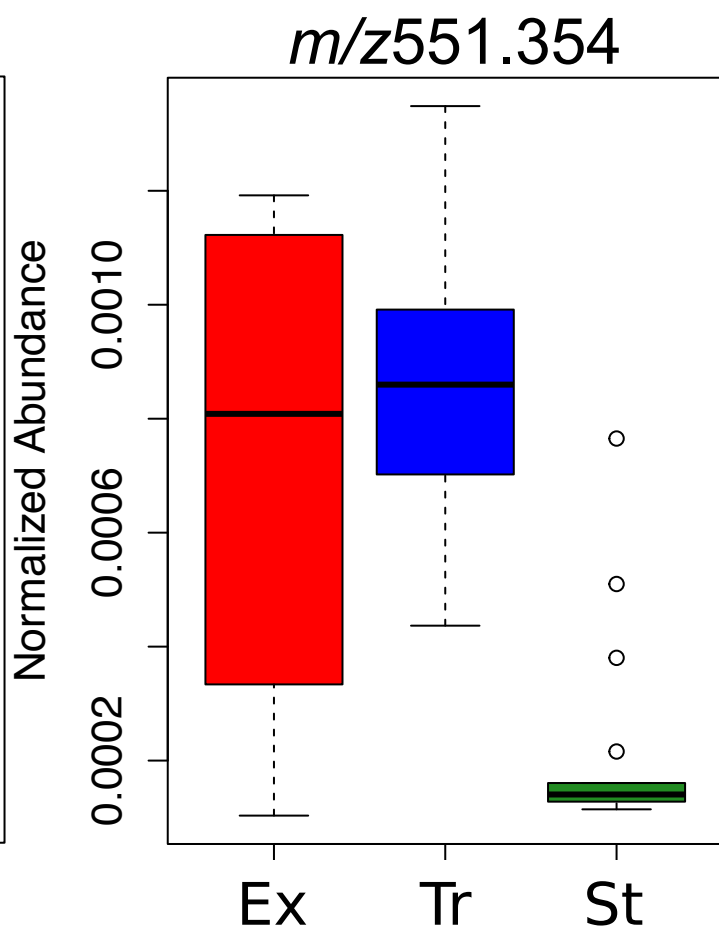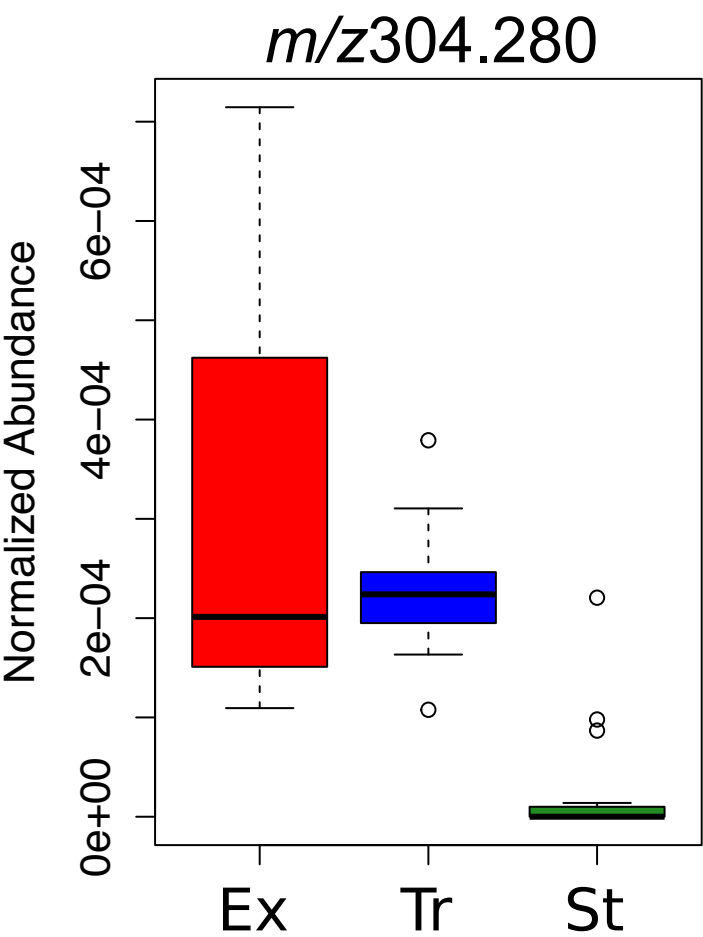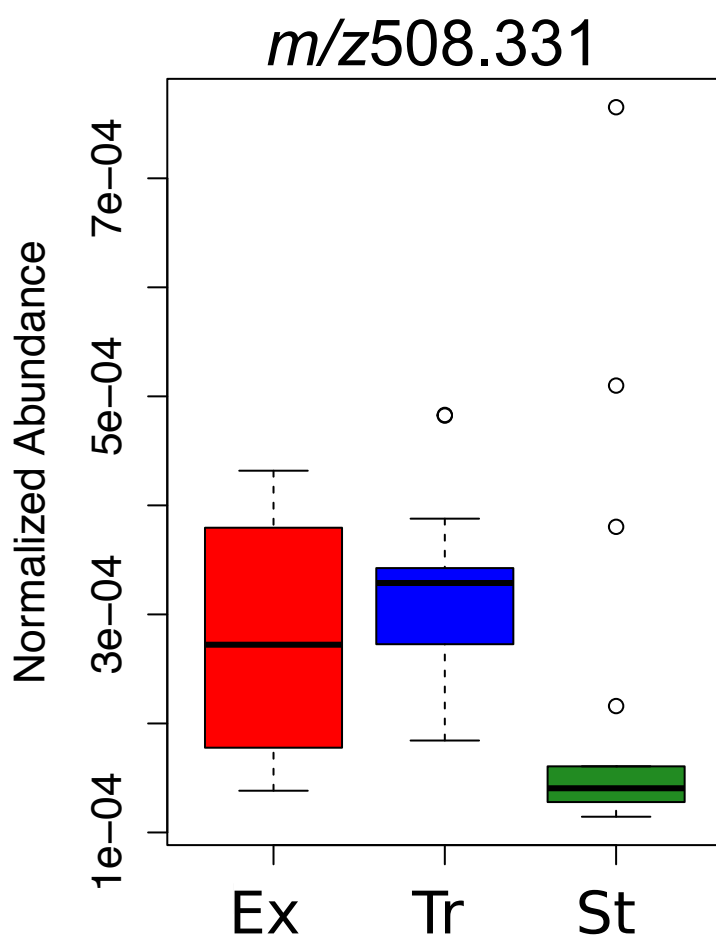

Supplement: Figure S4 [file peerj-04-2174-s004.pdf]

$m/z$ 442.317

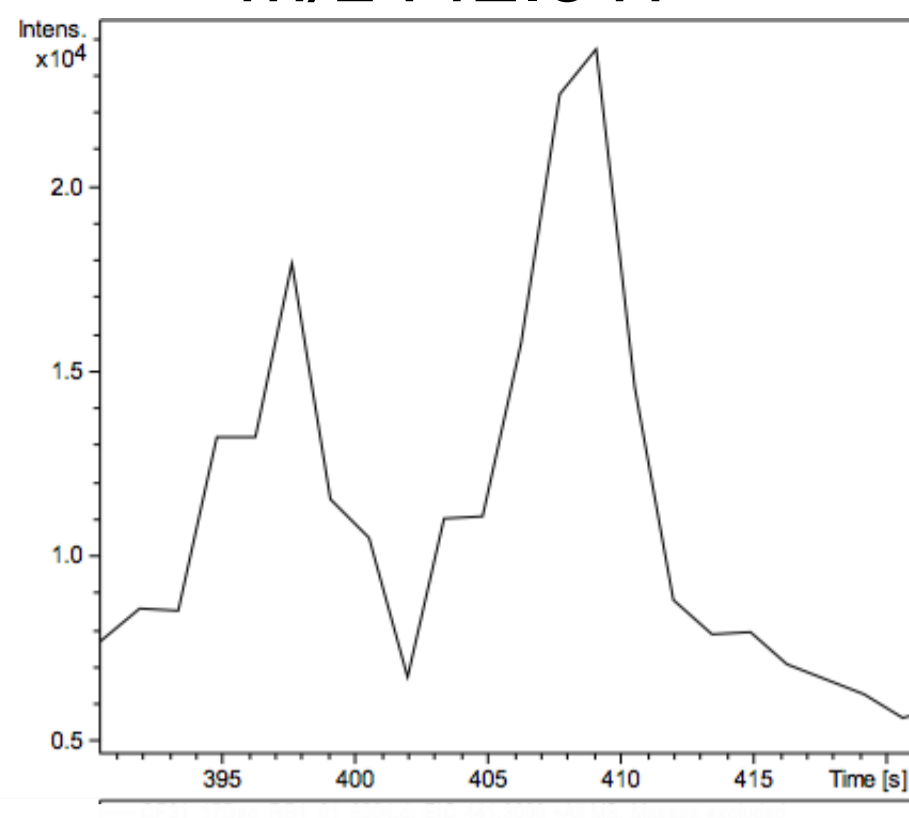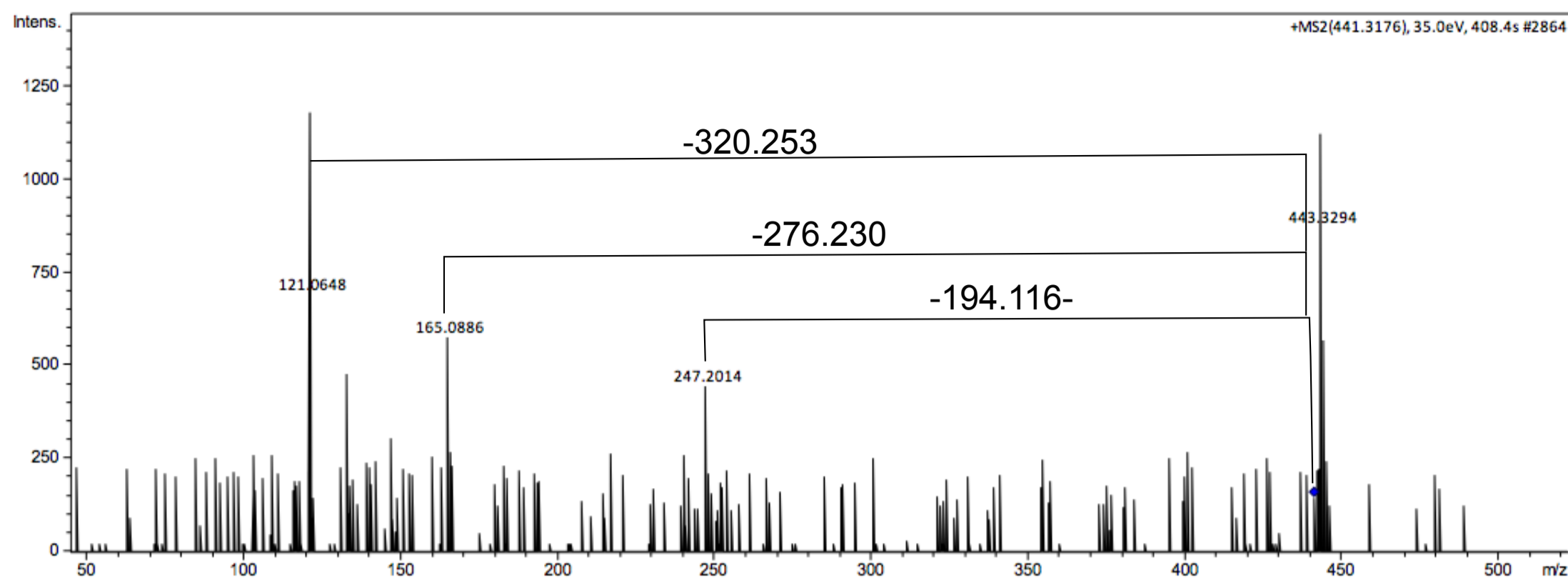

$m/z$ 304.281

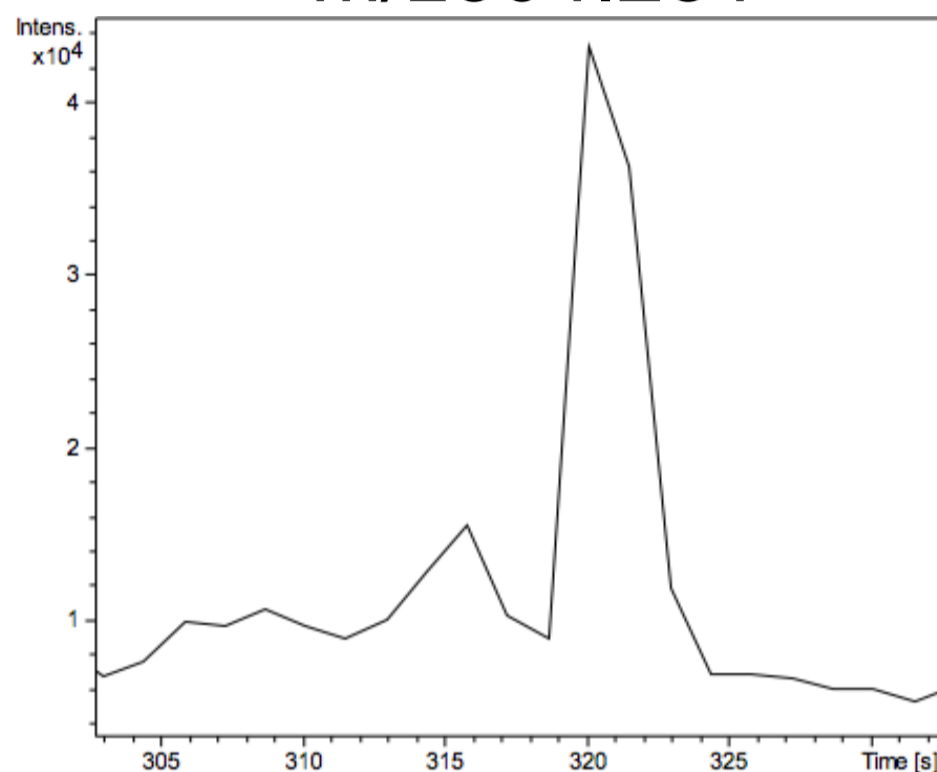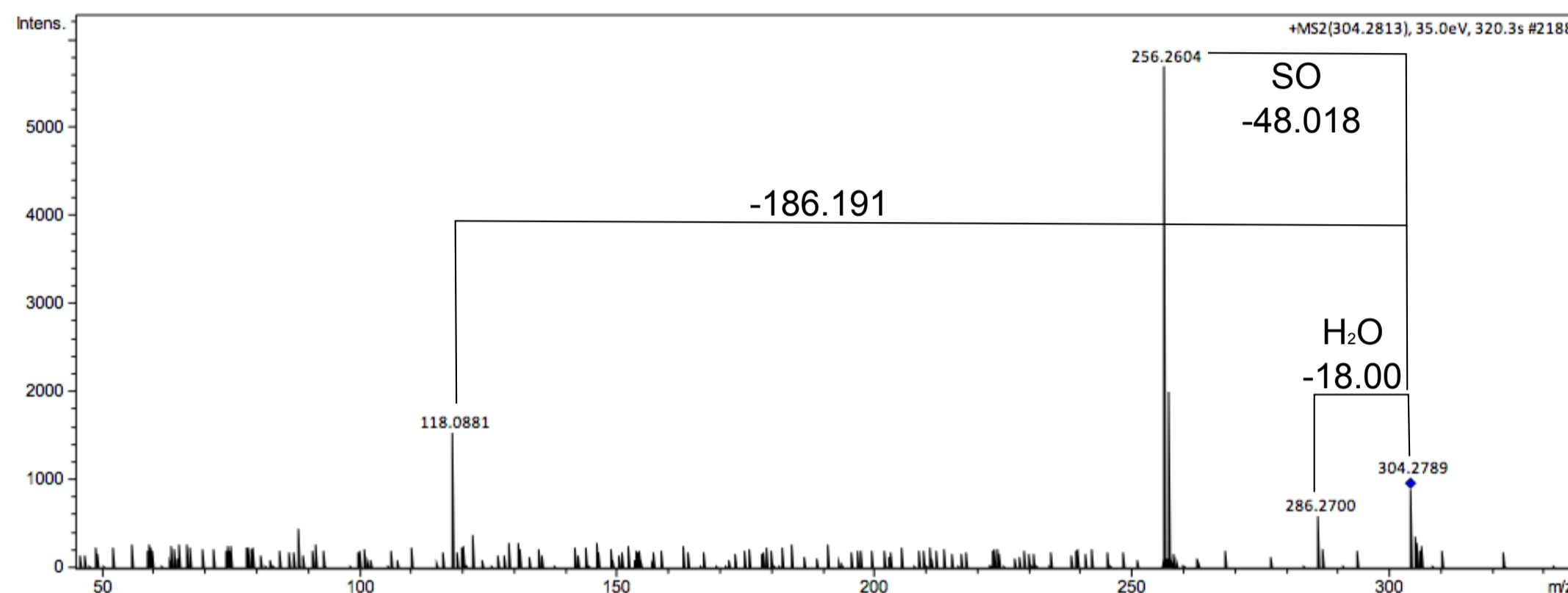

$m/z$ 508.374

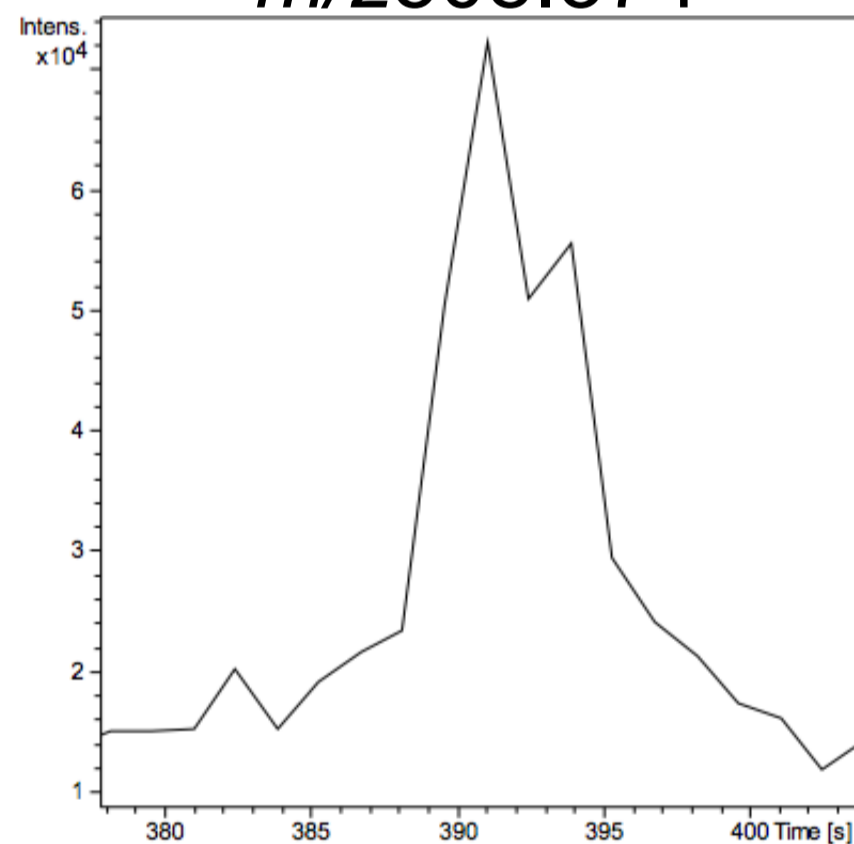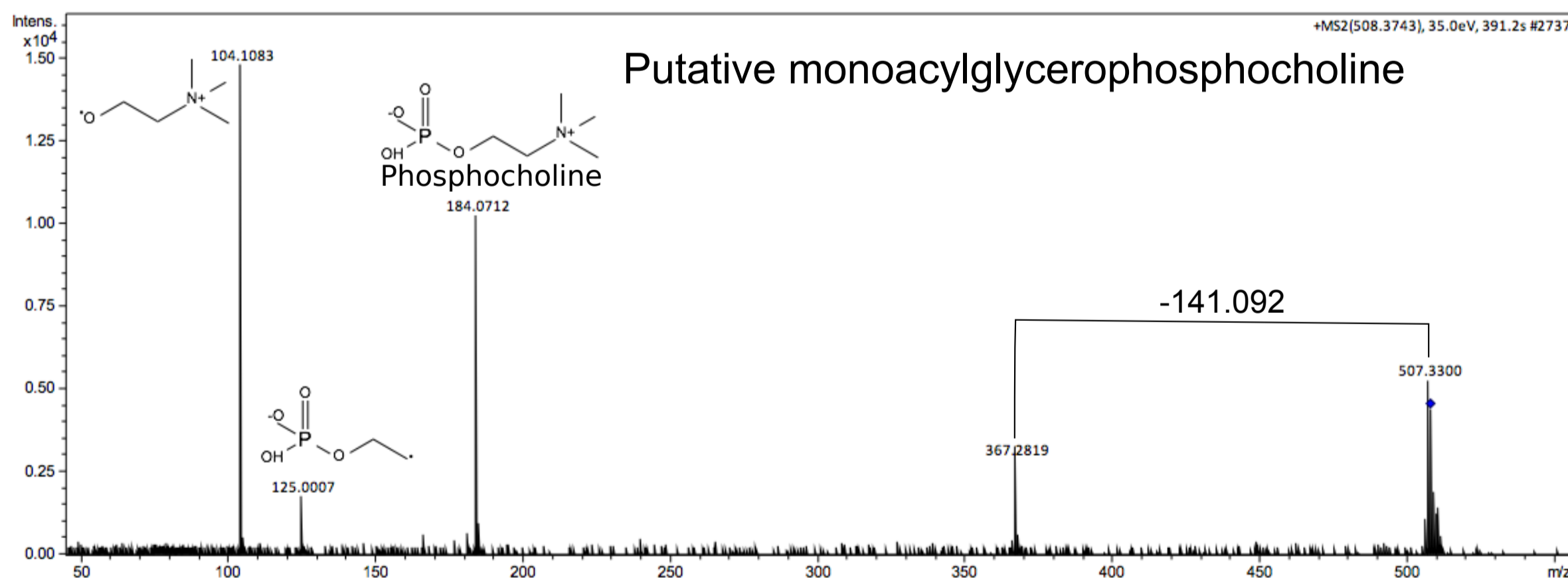

$m/z$ 551.354

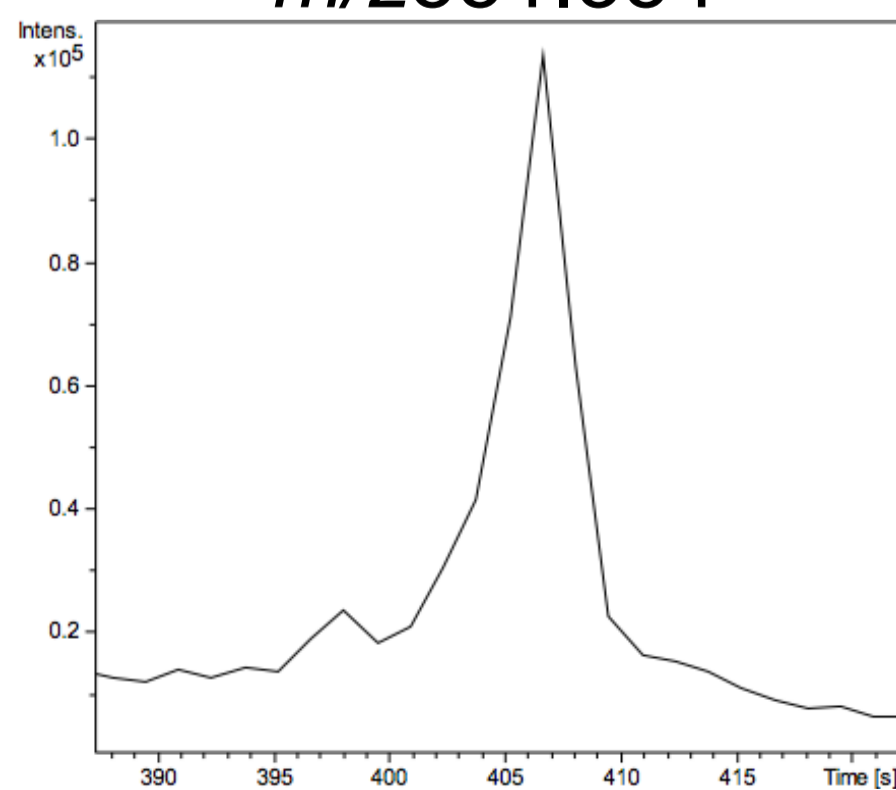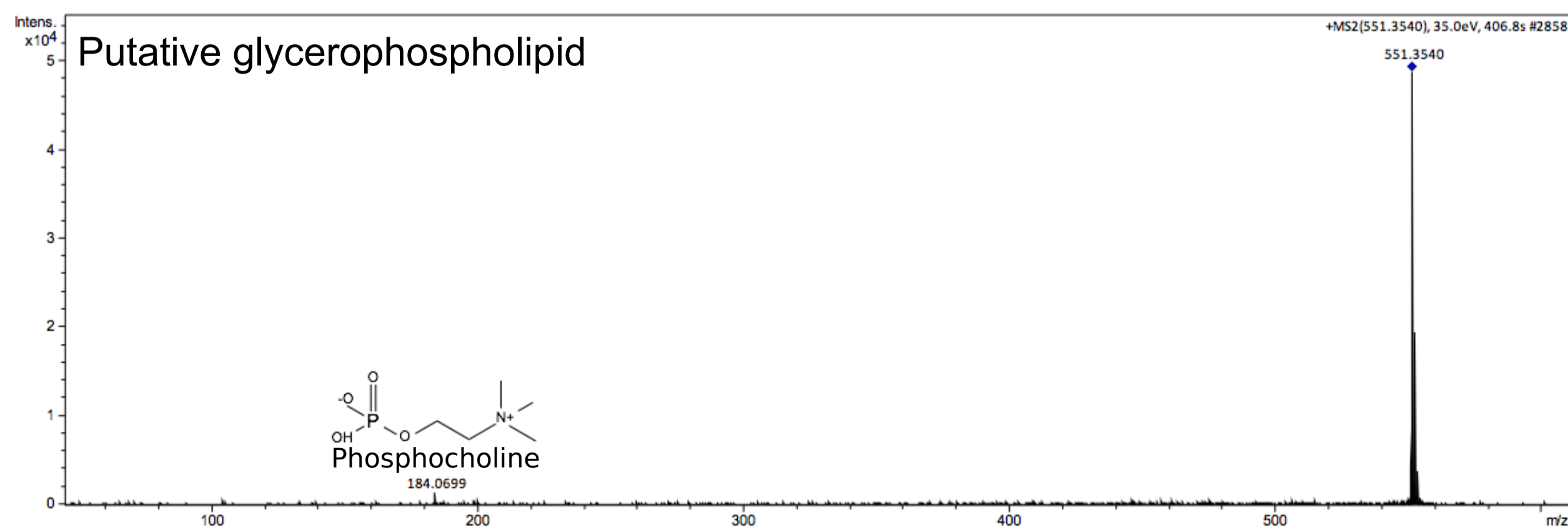

Supplement: Figure S5 [file peerj-04-2174-s005.pdf]
