# Supplementary material for: Metabolomics of pulmonary exacerbations reveals the personalized nature of cystic fibrosis disease
Source: PeerJ. 2016 Aug 11;4:e2174. doi: 10.7717/peerj.2174 (PMC4991883; doi:10.7717/peerj.2174)
Supplement: Table S1 — Patients and disease states for longitudinal sputum samples in this study. Table S2. Longitudinal sputum samples collected from patient CF1 in this study. [file peerj-04-2174-s006.docx]

Table S1. Patients and disease states for longitudinal sputum samples in this study.

| Dataset 1 | | Dataset 2 | | |  | | Overall | | | | | | Overall | | | | | |  |
| --- | --- | --- | --- | --- | --- | --- | --- | --- | --- | --- | --- | --- | --- | --- | --- | --- | --- | --- | --- |
| Patient | Clinical State | Patient | Clinical State | |  | Patient | | | | Samples | | Clinical State | | | Samples | |  |  |  |
| CF6 | Ex | CF5 | Ex | |  | 3 | | | | 3 | | Ex | | | 20 | |  |  |  |
| CF6 | Pt | CF5 | Ex | |  | 5 | | | | 5 | | Tr | | | 8 | |  |  |  |
| CF6 | St | CF5 | Ex | |  | 9 | | | | 3 | | Pt | | | 8 | |  |  |  |
| CF2 | Ex | CF5 | Pt | |  | 10 | | | | 3 | | St | | | 8 | |  |  |  |
| CF2 | Ex | CF5 | Pt | |  | 11 | | | | 5 | |  | | |  | |  |  |  |
| CF2 | Ex | CF9 | Pt | |  | 4 | | | | 5 | |  | | |  | |  |  |  |
| CF2 | Ex | CF9 | St | |  | 7 | | | | 3 | |  | | |  | |  |  |  |
| CF2 | St | CF9 | St | |  | 8 | | | | 4 | |  | | |  | |  |  |  |
| CF2 | Tr | CF10 | Ex | |  | 1 | | | | 4 | |  | | |  | |  |  |  |
| CF1 | Ex | CF10 | Pt | |  | 6 | | | | 3 | |  | | |  | |  |  |  |
| CF1 | St | CF10 | Tr | |  | 2 | | | | 6 | |  | | |  | |  |  |  |
| CF1 | St | CF11 | Ex | |  |  | | | |  | |  | | |  | |  |  |  |
| CF1 | Tr | CF11 | Ex | |  |  | | | |  | |  | | |  | |  |  |  |
| CF8 | Ex | CF11 | St | |  |  | | | |  | |  | | |  | |  |  |  |
| CF8 | Ex | CF11 | Tr | |  |  | | | |  | |  | | |  | |  |  |  |
| CF8 | Pt | CF11 | Tr | |  |  | | | |  | |  | | |  | |  |  |  |
| CF8 | Tr | CF3 | Ex |  | |  | | | | |  |  | | | |  |  |  |  |
| CF7 | Ex | CF3 | Ex |  | |  | | | | |  |  | | | |  |  |  |  |
| CF7 | Ex | CF3 | Ex |  | |  | | | | |  |  | | | |  |  |  |  |
| CF7 | Pt |  |  | |  | | |  |  | | |  | |  | | | |  | |
| CF4 | Ex |  |  | |  | | |  |  | | |  | |  | | | |  | |
| CF4 | Pt |  |  | |  | | |  |  | | |  | |  | | | |  | |
| CF4 | St |  |  | |  | | |  |  | | |  | |  | | | |  | |
| CF4 | Tr |  |  | |  | | |  |  | | |  | |  | | | |  | |
| CF4 | Tr |  |  | |  | | |  |  | | |  | |  | | | |  | |

Table S2. Longitudinal sputum samples collected from patient CF1 in this study.

| Counting Days | Clinical State | 16S data from (17) |
| --- | --- | --- |
| 1 | St |  |
| 84 | Ex |  |
| 98 | Tr |  |
| 118 | St |  |
| 420 | St |  |
| 509 | Ex | Y |
| 510 | Tr | Y |
| 511 | Tr | Y |
| 512 | Tr | Y |
| 513 | Tr | Y |
| 514 | Tr | Y |
| 515 | Tr | Y |
| 516 | Tr | Y |
| 517 | Tr | Y |
| 518 | Tr | Y |
| 519 | Tr | Y |
| 521 | Tr | Y |
| 522 | Tr | Y |
| 572 | St |  |
| 656 | Ex |  |
| 663 | Tr |  |
| 666 | Tr |  |
| 1085 | St |  |
| 1092 | St |  |
| 1093 | St |  |
| 1100 | St |  |
| 1108 | St |  |
| 1122 | St |  |
| 1130 | St |  |
| 1144 | St |  |
| 1151 | St |  |
| 1172 | St |  |
| 1186 | St |  |
| 1193 | St |  |
| 1222 | St |  |
| 1243 | St |  |
| 1523 | Ex |  |
