# Supplementary material for: Metabolomics of pulmonary exacerbations reveals the personalized nature of cystic fibrosis disease
Source: PeerJ. 2016 Aug 11;4:e2174. doi: 10.7717/peerj.2174 (PMC4991883; doi:10.7717/peerj.2174)
Supplement: Supplemental Information 1 [file peerj-04-2174-s007.docx]

**Supplementary Methods**

*Sample Collection:* The two sputum sample sets were stored differently, the first was kept on ice immediately after collection for DNA extraction in a previous study (Lim et al., 2014) and then frozen at -80°C after sample processing and the second sample set was stored immediately in liquid nitrogen. Due to the differences in sample collection, the global chemistry of these two samples was only compared within each collection. Samples were collected longitudinally under the following time point classification: *exacerbation* (Ex), at the presentation of a CFPE prior to antibiotic treatment, *treatment* (Tr), during antibiotic therapy within 24 h prior to a treatment change, *post-treatment* (Pt), within twelve hours of the cessation of antibiotic treatment, and *stable* (St), approximately 4 weeks after treatment when the patient was in a state of relative stability. All samples were collected within the requirements of the University of California at San Diego institutional review board (HRPP # 081500) and the San Diego State University Institutional review board (SDSU IRB 2121). Information about the prescribed antibiotics for CF1 was obtained through the patient clinical history from the attending clinician. 5 of these samples analyzed in this study were previously analyzed by metagenomic sequencing (Lim et al., 2013; Quinn et al., 2014).

An additional 4 sputum samples were collected to assess the technical variation in our extraction and analysis workflow. These sputum samples were collected using the same methods above and run under the same LC-MS/MS conditions described below, except 3 aliquots of one sample was extracted separately and then again on three successive days to assess the degree of variability and homogeneity within the sample and its analysis procedure (see supplemental results).

*LC-MS/MS.* The mass spectrometer was tuned using Tuning Mix ES-TOF (Agilent Technologies) at a 3 mL min^-1^ flow rate once a day during the run. For accurate mass measurements, lock mass internal calibration used a wick saturated with hexakis (1H,1H,3H-tetrafluoropropoxy) phosphazene ions (Synquest Laboratories, *m/z* 922.0098) located within the source. Chromatography was performed using a ThermoScientific UltraMate 3000 Dionex UPLC. A sample volume of 30 μL of the ethyl acetate/methanol extracts were separated using a Kinetex 2.6 μm C18 (30 x 2.10 mm) UPLC column. The flow rate was 0.5 mL min^-1^. Full scan MS spectra (*m/z* 50 – 2000) were acquired in the qTOF and the top ten most intense ions in a particular scan were fragmented using collision induced dissociation at 35 eV for +1 ions and 25 eV for +2 ions in the collision cell. Automatic exclusion was used such that an ion was fragmented upon its first detection, then fragmented twice more, but not again unless its intensity was 2.5x the previous fragmentation. This exclusion method was removed after 30 seconds and the mass spectrometer would repeat its ion detection and automatic exclusion cycle. All metabolomic data is available at gnps.ucsd.edu under massive accession numbers: MSV000079104, MSV000079443, and MSV000079444.

*Metabolome Generation:* For comparison of overall metabolome similarity the sample sets were processed and analyzed separately to ensure that global metabolome comparisons were only attempted on samples that were run on the LC-MS/MS on the same day to avoid batch effects observed at the level of global comparison. The ‘find molecular features’ algorithm was used to identify molecular features with the following parameters: a signal-to-noise ratio of 5, a correlation coefficient threshold of 0.7, a minimum compound length of 8 and a smoothing width of 2. The Bruker® ProfileAnalysis bucket table was calculated from 60 to 800 s using the time alignment feature with targeting ions of *m/z* 150 to 1500 and with a bucket table value count greater than 2. The metabolome was normalized by dividing each molecular feature abundance by the total ion abundance of its sample.

For biomarker detection based on ion abundance, a retention time alignment procedure was used for the 2012, 2014, and CF1 longitudinal data sets do identify the same ions across the batches. These sample sets were all run using the same LC-MS/MS methods. By utilizing a strict tolerance (< 15 ppm) on *m/z* value variation coupled with more flexible non-parametric procedures for retention time drift and bias correction, mutual spectral features across the different metabolomic data sets acquired at different times and from different patients were maximized. Raw spectral peak data was initially acquired via the XCMS CentWave algorithm (Tautenhahn, Böttcher & Neumann, 2008) for all samples (peak width min=10s, max=25s; consecutive scan limit = 15ppm). The largest sample set was designated as the “master” set, to be used as a reference point by which data from all other sets was subsequently aligned to. Batch level variations in mass accuracy or potential instrument miscalibration resulting in biased m/z values were corrected for via localized re-averaging of raw mass values. Multi-set retention time alignment was conducted via “endogenous anchors,” which are the most highly recurrent spectral features (>90% of samples) in the master set, and therefore the most likely features to appear in all other sets. Endogenous anchors act as reference points for aligning the retention times of spectral features in a given sample to the master set, and is capable of accounting for drift and per-batch bias in a robust non-parametric manner.

*16S rDNA Sequencing and Metabolome Correlations*: Only correlation pairs whose Pearson’s *r* were below a permutation test p-value of 0.05 were included in the network. The *r* was used as the ‘edge’ list in Cytoscape® with the bacterial genera as the central nodes and all molecular features correlated with that genera connected by an edge. The thickness of the edge was scaled to the strength of *r*. Molecular feature nodes in the network were colored according to which bacteria they were correlated with.

*Metabolome Annotation:* Molecular networks were built with a cosine cutoff of 0.65 a minimum matched peaks of 4 and a topK value of 10. Networks were visualized using the Cytoscape® software (Shannon et al., 2003). GNPS was searched using the following molecular networking parameters: a minimum matched peak threshold of 3, cosine score threshold of 0.7 and a minimum cluster size of 2. Putative annotation of other metabolites was done manually by comparing to known spectra in the METLIN database (Smith et al., 2005). Annotated metabolites were visualized in the Cystoscape® network to identify their correlations with particular bacterial genera. Standards of all known molecules reported in this data set were separately ran on the same instrument and uploaded to GNPS as a spectral library prior to this study. As such, all annotations of metabolites in this manuscript are at level two according to the proposed minimal reporting standards in metabolomics. (Sumner et al., 2007).

Lim YW., Schmieder R., Haynes M., Willner D., Furlan M., Youle M., Abbott K., Edwards R., Evangelista J., Conrad D., Rohwer F. 2013. Metagenomics and metatranscriptomics: Windows on CF-associated viral and microbial communities. *J. Cyst. Fibros.* 12:154–164.

Lim YW., Evangelista JS., Schmieder R., Bailey B., Haynes M., Furlan M., Maughan H., Edwards R., Rohwer F., Conrad D. 2014. Clinical insights from metagenomic analysis of cystic fibrosis sputum. *Journal of clinical microbiology* 52:425–457.

Quinn RA., Lim YW., Maughan H., Conrad D., Rohwer F., Whiteson KL. 2014. Biogeochemical forces shape the composition and physiology of polymicrobial communities in the cystic fibrosis lung. *mBio* 5.

Shannon P., Markiel A., Ozier O., Baliga NS., Wang JT., Ramage D., Amin N., Schwikowski B., Ideker T. 2003. Cytoscape: a software environment for integrated models of biomolecular interaction networks. *Genome research* 13:2498–504.

Smith CA., O’Maille G., Want EJ., Qin C., Trauger SA., Brandon TR., Custodio DE., Abagyan R., Siuzdak G. 2005. METLIN: a metabolite mass spectral database. *Therapeutic drug monitoring* 27:747–51.

Sumner LW., Amberg A., Barrett D., Beale MH., Beger R., Daykin CA., Fan TW-M., Fiehn O., Goodacre R., Griffin JL., Hankemeier T., Hardy N., Harnly J., Higashi R., Kopka J., Lane AN., Lindon JC., Marriott P., Nicholls AW., Reily MD., Thaden JJ., Viant MR. 2007. Proposed minimum reporting standards for chemical analysis Chemical Analysis Working Group (CAWG) Metabolomics Standards Initiative (MSI). *Metabolomics : Official journal of the Metabolomic Society* 3:211–221.

Tautenhahn R., Böttcher C., Neumann S. 2008. Highly sensitive feature detection for high resolution LC/MS. *BMC bioinformatics* 9:504.
