# Supplementary material for: Metabolomics of pulmonary exacerbations reveals the personalized nature of cystic fibrosis disease
Source: PeerJ. 2016 Aug 11;4:e2174. doi: 10.7717/peerj.2174 (PMC4991883; doi:10.7717/peerj.2174)
Supplement: Supplemental Information 2 [file peerj-04-2174-s008.pdf]

The ISME Journal (2015) 9, 1024–1038
© 2015 International Society for Microbial Ecology All rights reserved 1751-7362/15
www.nature.com/ismej

ORIGINAL ARTICLE

A Winogradsky-based culture system shows an association between microbial fermentation and cystic fibrosis exacerbation

This article has been corrected since Advance Online Publication and a corrigendum is also printed in this issue

Robert A Quinn[1], Katrine Whiteson[1], Yan-Wei Lim[1], Peter Salamon[2], Barbara Bailey[2], Simone Mienardi[3], Savannah E Sanchez[1], Don Blake[3], Doug Conrad[4] and Forest Rohwer[1]
[1]*Department of Biology, San Diego State University, San Diego, CA, USA;* [2]*Department of Mathematics and Statistics, San Diego State University, San Diego, CA, USA;* [3]*Department of Chemistry, University of California, Irvine, CA, USA and* [4]*Department of Medicine, University of California, San Diego, CA, USA*

There is a poor understanding of how the physiology of polymicrobial communities in cystic fibrosis (CF) lungs contributes to pulmonary exacerbations and lung function decline. In this study, a microbial culture system based on the principles of the Winogradsky column (WinCF system) was developed to study the physiology of CF microbes. The system used glass capillary tubes filled with artificial sputum medium to mimic a clogged airway bronchiole. Chemical indicators were added to observe microbial physiology within the tubes. Characterization of sputum samples from seven patients showed variation in pH, respiration, biofilm formation and gas production, indicating that the physiology of CF microbial communities varied among patients. Incubation of homogenized tissues from an explant CF lung mirrored responses of a *Pseudomonas aeruginosa* pure culture, supporting evidence that end-stage lungs are dominated by this pathogen. Longitudinal sputum samples taken through two exacerbation events in a single patient showed that a two-unit drop in pH and a 30% increase in gas production occurred in the tubes prior to exacerbation, which was reversed with antibiotic treatment. Microbial community profiles obtained through amplification and sequencing of the 16S rRNA gene showed that fermentative anaerobes became more abundant during exacerbation and were then reduced during treatment where *P. aeruginosa* became the dominant bacterium. Results from the WinCF experiments support the model where two functionally different CF microbial communities exist, the persistent Climax Community and the acute Attack Community. Fermentative anaerobes are hypothesized to be the core members of the Attack Community and production of acidic and gaseous products from fermentation may drive developing exacerbations. Treatment targeting the Attack Community may better resolve exacerbations and resulting lung damage.
The ISME Journal (2015) **9,** 1024–1038; doi:10.1038/ismej.2014.234; published online 16 December 2014

Introduction

The cystic fibrosis (CF) lung contains a complex community of interacting microbes and viruses (Willner *et al.*, 2009; Sibley and Surette, 2011; Yang *et al.*, 2011; Delhaes *et al.*, 2012; Lim *et al.*, 2012; Rabin and Surette, 2012; Zhao *et al.*, 2012; Lim *et al.*, 2013). These communities shape microenvironments within the lung with their own biological, chemical and physical characteristics. These microenvironments allow for the application of fundamental principles of microbial ecology (Yang *et al.*, 2011; Conrad *et al.*, 2012). For example, synergistic and antagonistic behavior between different CF pathogens has been described (Duan *et al.*, 2003; Harrison, 2007; Sibley *et al.*, 2008; Mitchell *et al.*, 2010; Yang *et al.*, 2011; Venkataraman *et al.*, 2014), and the microbial community has been proposed to contain stable and disturbed states, much like those in classical ecology (Conrad *et al.*, 2012). Known chemical characteristics of the CF lung include lowered pH (Tate *et al.*, 2002; Pezzulo *et al.*, 2012), low oxygen tensions (Worlitzsch *et al.*, 2002) and high levels of amino acids, nitrate, iron and phenazines (Grasemann *et al.*, 1998; Jones *et al.*, 2000; Palmer *et al.*, 2007; Ghio *et al.*, 2012; Hunter *et al.*, 2012). In the environment, microbial communities are known to stratify based on oxygen and other chemical gradients (Fenchel and Finlay, 2008; Morris and Schmidt, 2013). CF mucus also contains oxygen gradients (Worlitzsch *et al.*, 2002; Morris and Schmidt, 2013), suggesting that microbes may stratify similarly. Thus the micro-environment of a mucus-filled bronchiole can be considered

Correspondence: RA Quinn, Department of Biology, San Diego State University, 5500 Campanile Drive, San Diego, CA 92182, USA.
E-mail: quinnr1234@gmail.com
Received 16 July 2014; revised 24 October 2014; accepted 4 November 2014; published online 16 December 2014

analogous to the Winogradsky column, developed by Sergei Winogradsky to study the stratification of microbial communities in sediment based on chemical and oxygen gradients (Winogradsky, 1897). Effective study of microbial behavior in CF requires a re-formation of the chemical and physical environment known to exist *in vivo* and the application of microbial ecology approaches.

CF patients undergo intermittent pulmonary exacerbations (CFPEs), a condition requiring aggressive treatment, including antibiotics (Stenbit and Flume, 2011). CFPEs cause damage to the lungs and are associated with patient health decline (De Boer *et al.*, 2011). The microbial behavior during CFPE is poorly understood. Although metagenomic studies have revealed aspects of the core physiology of CF microbes containing both respiratory and fermentative metabolisms (Lim *et al.*, 2012; Quinn *et al.*, 2014), little is known about microbial physiology during exacerbation events. Although some studies report that the microbial community changes as exacerbations develop (Carmody *et al.*, 2013; Twomey *et al.*, 2013), others provide evidence for relative stability (Stressmann *et al.*, 2011; Price *et al.*, 2013). A better understanding of the microbial community dynamics and physiology through CFPE is critical to understand how this community contributes to disease flares.

Current methods for monitoring CF infections rely on bacterial culture techniques using selective media for known CF-associated pathogens. Antibiotic treatment regimes for exacerbations are determined based on these culture results plus antimicrobial susceptibility testing (Hauser *et al.*, 2011). This treatment strategy is inherently biased towards aerobic microbes that grow on media selected by clinical microbiologists (Burns and Rolain, 2014). There is growing evidence that CF microbes survive in a low oxygen environment and many grow anaerobically deep in the mucus plugs of CF lungs (Worlitzsch *et al.*, 2002, 2009; Bjarnsholt *et al.*, 2009; Lipuma, 2010). Thus conventional culture on microbiological media misses important components of the CF microbial community that may contribute to exacerbations and provides antibiotic resistance profiles from microbes not grown in a state in which they cause disease. For example, *Pseudomonas aeruginosa* has very different antimicrobial susceptibilities under aerobic, anaerobic or biofilm growth (Hill *et al.*, 2005; King *et al.*, 2010). Growth of clinical samples in an environment more similar to the CF lung would provide better information and more predictable treatment outcomes.

Here we describe a novel, highly tractable, culture-based system that provides rapid information regarding the physiology of CF microbial communities. The system is called the Winogradsky cystic fibrosis system (WinCF system), because it is based on the principles of the Winogradsky column. Microbial growth in the tubes creates steep oxygen gradients mimicking the environment in CF mucus plugs (Worlitzsch *et al.*, 2002). This system was applied to study pure cultures of CF isolates, sputum samples and tissue homogenates from an explant lung. The physiochemical variables monitored in the WinCF system include changes in pH, respiratory activity, biofilm growth and gas production due to microbial activity. The results showed patient-specific differences in microbial physiology and demonstrated a shift to fermentation at exacerbation.

Materials and methods

Strains and culture conditions
Bacterial strains of non-mucoid *P. aeruginosa* (PAnmFLR01), mucoid *P. aeruginosa* (PAmFLR02) and *Stenotrophomonas maltophilia* (SmFLR01) were isolated from CF sputum collected at the UCSD adult CF clinic (La Jolla, CA, USA). *Streptococcus salivarius* (SxFLR02) was isolated from the sputum of patient CF1 using Columbia naladixic acid agar supplemented with 5% sheep's blood. All isolates were frozen in 20% glycerol after initial isolation and then maintained on Todd Hewitt Agar at 37 °C. For inoculation into the WinCF system, each culture was grown to an optical density of 0.2 at 37 °C in Todd Hewitt broth, pelleted by centrifugation and resuspended in an equal volume of phosphate-buffered saline.

Sample collections
Samples were collected in compliance with the University of California Institutional Review Board (HRPP 081500) and San Diego State University Institutional Review Board (SDSU IRB no. 2121) requirements. Single cross-sectional sputum samples were collected from seven CF and six non-CF volunteers. CF volunteers were identified based on CFTR (cystic fibrosis transmembrane conductance regulator) genetics and/or sweat test; non-CF volunteers were not known to have any CFTR mutations. Longitudinal sputum samples were collected from a single patient through time for a total of 26 sputa over 198 days and two separate exacerbation events. The decision to treat for an exacerbation was determined by the treating clinician at the time of sputum sampling. For CFPE1, the patient presented with five of the Fuchs criteria (Fuchs *et al.*, 1994), including shortness of breath, chest pain, increased cough, increased sputum production, anorexia and a decrease in lung FEV_1% of 23%, and was diagnosed and treated for a severe exacerbation with intravenous tobramycin, imipenem, linezolid and trimethoprim. For CFPE2, the patient presented with three of the Fuchs criteria, including increased sputum production, expectoration of blood and increased sinus pressure, was diagnosed with a mild exacerbation and treated with the oral antibiotics ciprofloxacin and doxycycline. All samples were collected by hypertonic saline induction and expectorated directly into a sterile sputum cup. The sample was immediately homogenized

according to a previously published method (Lim *et al.*, 2012). Lung tissue sections were excised from an explanted left lung of a CF patient about to receive a lung transplant. Tissue sections approximately 1 cm^2 were homogenized using a hand-held homogenizer in the presence of sterile phosphate-buffered saline. All sputum samples and tissue homogenates were kept on ice until inoculation into the WinCF system within 24 h of sample collection.

Inoculation of WinCF system

Artificial sputum medium (Fung *et al.*, 2010) contained 20 mg ml^{-1} pig stomach mucin (Sigma-Aldrich, St Louis, MO, USA), 1.4 mg ml^{-1} DNA, (Salmon Sperm DNA, Sigma-Aldrich), 2.2 mg ml^{-1} KCl, 5 mg ml^{-1} NaCl, 7.225 × amino acids (Sigma-Aldrich), 14.45 × MEM-Non-Essential amino acids (Sigma-Aldrich) and 0.005% egg yolk emulsion (Dalynn Biologicals, Calgary, AB, Canada). The media was modified to include 3 μg ml^{-1} ferritin as an alternate iron source to more appropriately reflect the conditions of the CF lung (Stites *et al.*, 1998; Reid *et al.*, 2004; Ghio *et al.*, 2012; Hare *et al.*, 2012). Other modifications included the absence of bovine serum albumin and an increase to 20 mg ml^{-1} porcine stomach mucin to better reflect our experience with CF sputum consistency. The media pH was initially 7.4 ± 0.1. The media will hereafter be referred to as ASMRQ and its constituents are listed in Supplementary Table S1. Glass capillary tubes measuring 75 mm in length with 1.4 mm internal diameter (Thermo Fischer Scientific Inc., San Diego, CA, USA) were used for the WinCF system. Chemical indicators were added to the ASMRQ media (Supplementary Table S1), and a sample volume of 1/10 total media volume was used for inoculation into the WinCF system. For pH monitoring, the media contained 40 μg ml^{-1} Phenol Red and 40 μg ml^{-1} Bromocresol Purple (Yao and Byrne, 2001), and for redox activity the media contained 1 × tetrazolium dye Mix A (Biolog Inc., Hayward, CA, USA). To observe biofilm growth or plugging due to cell aggregation, a final concentration of 0.004% w/v Coomassie Brilliant Blue was used. No indicators were added to the tubes used for the measurement of gas bubbles. The mixtures were vortexed, and the capillary tubes were filled by capillary action to approximately 65 μl and plugged using an ethanol-sterilized capillary tube sealant (Fisherbrand Hemato-Seal, Thermo-Fisher, San Diego, CA, USA) at one end (Supplementary Figure S1). The tubes were prepared in triplicate for each biochemical variable and incubated in 15-ml falcon tubes stuffed with wet paper towel (Supplementary Figure S1). All tubes were incubated at 37 °C for 48 h vertically, except for the gas bubble tubes, which were incubated horizontally.

Capillary tube imaging

After 48 h of incubation, the capillary tubes were laid horizontally on two plastic holders 6 cm apart with 2 mm grooves on top of a Logan 5.5 × 9 $inches^2$ light box (Supplementary Figure S1). A Canon EOS Rebel T3 camera (Canon U.S.A Inc., Melville, NY, USA) was fixed at a height of 25 cm for imaging of tubes. Images were manually focused, auto adjusted for ISO and captured in the dark. Alignment was done using vertical lines in the camera view and the sides of a single capillary tube. A set of phosphate buffers was prepared at pH 5.0, 5.5, 6.0, 6.5, 7.0, 7.5 and 8.0 to serve as the pH standards (Supplementary Figure S1). Each buffer was inoculated into a WinCF tube as previously described with the same concentrations of pH indicator dyes, except that these tubes were plugged at both ends. The pH of the sample tubes was measured based on colorimetric comparison to the pH standards (Supplementary Figure S1). Images were saved as JPEG format and imported into MATLAB for quantitative image analysis.

The oxygen gradient was visualized using a planar fluorescent ruthenium oxygen optode sheet (Haas *et al.*, 2013), which was cut into thin strips to fit into a capillary tube (Figure 1b). The strips were inserted after the WinCF tubes were prepared and incubated at 37 °C for 24 h. The tubes were imaged under Rebel Royal Blue light emitting diodes as described in Haas *et al.* (2013).

Image analysis

Image analysis was performed using MATLAB on cropped images such as that shown in Supplementary Figure S1. The image was segmented into individual tubes so that each tube represented a rectangle of red/green/blue values. For all but the gas tube analyses, these rectangles were averaged across the tube to a red/green/blue signal as a function of length along the tube. The pH tube analyses were based on interpolation of the average hue in the pH tubes compared with the hues of the standard tubes (Supplementary Figure S1). The redox tubes are a measure of an increase in purple color due to reduction of the tetrazolium dye in the tube. To compare redox across samples, a sterile tube was incubated and its level of color saturation from the MATLAB output was measured. All samples were then compared with this level by subtracting the mean saturation of each tube from the average of a sterile tube to get the change in redox, which was then multiplied by 100 to report the relative change in redox saturation as a percentage. The plugging tube analyses measured total length of the plugs as a fraction of the filled tube length. Similarly, gas tube analyses reported fraction tube length taken up by bubbles.

16S rRNA gene sequencing

The contents of the capillary tubes after incubation were removed with a syringe and 25-gauge needle

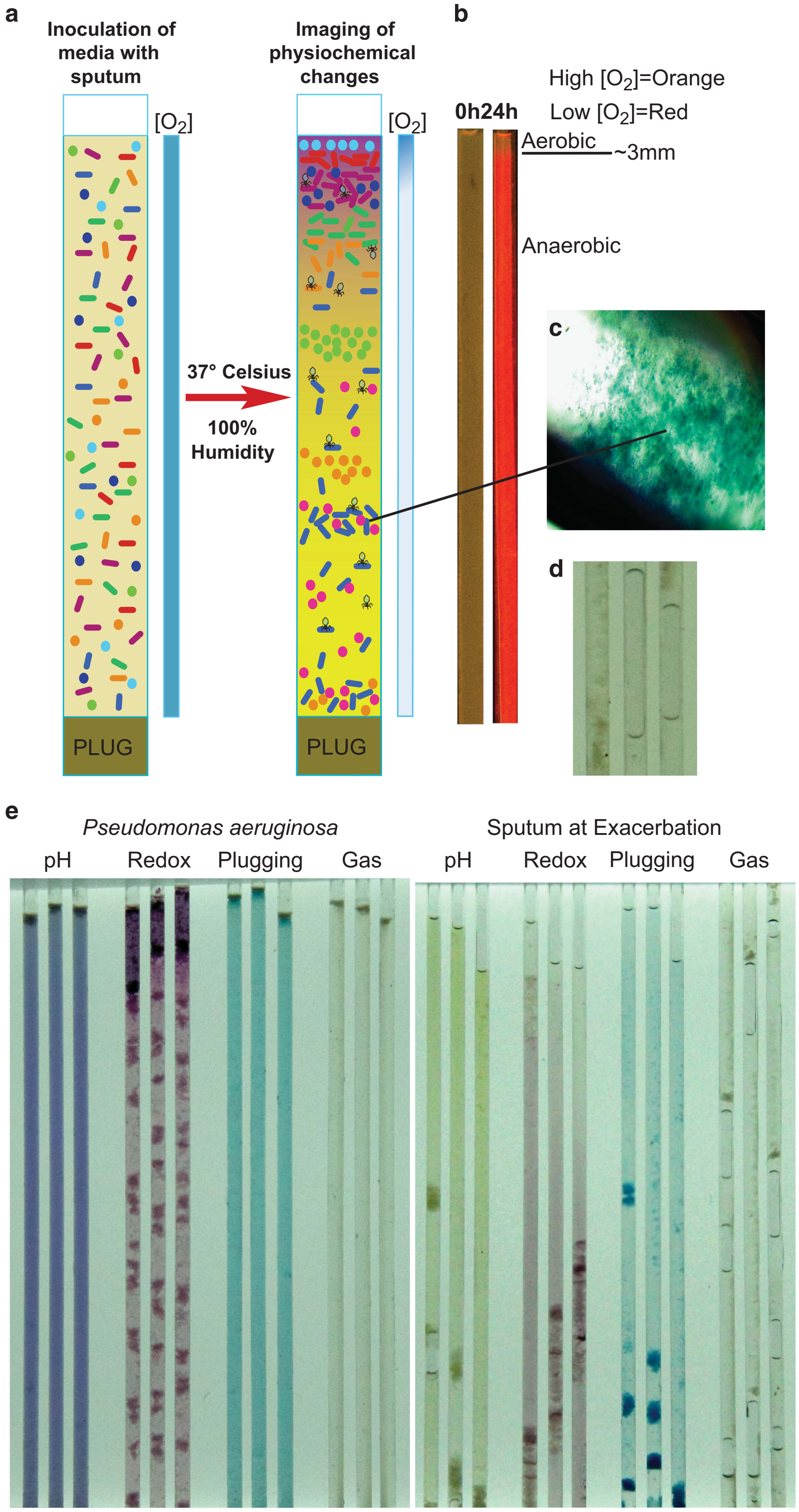

Figure 1 (**a**) Schematic of the WinCF model showing setup and principles of microbially induced physiochemical changes after incubation. (**b**) Fluorescent ruthenium oxygen optode before and after incubation with a pure culture of *P. aeruginosa* in the WinCF model showing removal of oxygen. (**c**) 40 × magnified capillary tube plug. (**d**) Capillary tube gas bubbles. (**e**) WinCF model image of a pure culture of *P. aeruginosa* (left panel) and an exacerbation sputum sample (right panel) showing the different physiochemical responses.

and immediately placed into 200 μl of the Trizol reagent (Life Technologies, Grand Island, NY, USA). Frozen sputum samples were thawed, and 200 μl of the sample was immediately placed into 500 μl of the Trizol reagent. DNA extraction was done according to the manufacturer's protocol (Life Technologies). Total DNA was sent to the Research Technology Support Facility Genomics Core at Michigan State University for 16S rRNA gene amplicon sequencing. The V4 region (515F/806R) of the bacterial 16S rRNA gene was amplified using universal primers (Caporaso *et al.*, 2011). After cleanup and quality control, the pooled libraries were sequenced on an Illumina MiSeq instrument using a standard MiSeq v2 reagent kit in a paired end 2×250 bp read format. The 20 million paired-end reads were preprocessed with PrinSeq (Schmieder and Edwards, 2011) (Supplementary Table S6). The resulting 5.3 million paired-end reads from 93 samples were made into individual contigs and analyzed using a new mothur standard operating procedure designed for MiSeq data (Kozich *et al.*, 2013). Clusters of operational taxonomic units with 97% sequence identity were formed, and their taxonomy was assigned using the silva database (Quast *et al.*, 2013) with Ribosomal Database Project taxonomy (Cole *et al.*, 2005). Microbial communities were compared using the random forest algorithm from the R statistical software package 'random forest'. Partitioning around mediods clustering was used to identify statistically strong clusters in the data.

Headspace gas capture and gas chromatography/mass spectrometry
The WinCF system capillaries were inoculated with a sputum sample that produced gas bubbles and was taken 2 days after oral antibiotic treatment began for CFPE2 (day 195 in Figures 3 and 5) and compared with sterile tubes containing the same ASMRQ media. A modified bioreactor and analytical gas chromatography and mass spectrometry system was used for headspace gas collection (Umber *et al.*, 2013), where replicates of six individual tubes were placed directly inside three separate glass collection containers (triplicate) and incubated at 37 °C for 24 h to collect headspace gas. Headspace gas was directly injected into a gas chromatography and mass spectrometry system and analyzed as described in Whiteson *et al.* (2014), Shin *et al.* (2009), Umber *et al.* (2013) and Colman *et al.* (2001). The area under the curve of selected volatile organic compounds was called manually and converted to parts-per-trillion where possible for the quantification of gases. Area under the curve for each gas was averaged from the triplicate samples, and a permutation test was used to identify significantly elevated gases from the media control, to avoid the assumption of normality in data distribution.

Results

Characteristics of the WinCF system
Inoculation of the capillary tubes with a non-mucoid *P. aeruginosa* strain initially created an aerobic environment at time 0 h (Figure 1b). After a 24-h incubation at 37 °C, the tubes were depleted of oxygen, resulting in a tight gradient at the open end of the tube showing oxygen penetration into the first 3 mm of media (Figure 1b). Thus, aerobic, microaerophillic and anaerobic microenvironments were created, with the majority of the media depleted of oxygen. At the time of inoculation, the media and chemical variables measured in the WinCF system, including pH, respiration, plugging and gas production, were homogenous throughout the tubes. After incubation of sterile tubes, the colorimetric indicators remained homogenous with few changes observed (Supplementary Figure S2a). Incubation with sputum or bacteria for at least 24 h induced drastic changes in the tube structure, and the chemical indicators were no longer homogenous (Figure 1). In the Coomassie Brilliant Blue-stained tubes, biomass accumulated at particular positions along the tube length, hereafter referred to as 'plugging'. Plugging appeared as particulates aggregated in the tube and suspended somewhere along its length (Figure 1c). When the WinCF tubes were incubated horizontally, gas bubbles of varying size occasionally accumulated (Figure 1d). Other observations included color changes in the pH and redox tubes and occasional fungal hyphae at the open end of the tube (Supplementary Figure S2c). The WinCF changes were most evident after a 48-h incubation. Samples were incubated in WinCF for a total of 5 days, but little change was observed after 48 h, if any change was observed; it was an intensification of the 48-h characteristics already established.

Behavior of pure cultures in WinCF system
A pure culture of non-mucoid *P. aeruginosa* strain (PAnmFLR01) had a particularly marked response in the WinCF system. This strain raised the pH of the media to 7.7, triggered large amounts of tetrazolium reduction (77% relative increase from sterile tube), particularly in the aerobic portion of the tube, plugged the entire tube with biomass and did not produce gas (Figure 1e and Table 1). A mucoid isolate of *P. aeruginosa* produced a similar response but without the extensive plugging of the tubes. The other CF pathogens tested (*S. maltophilia*, *S. salivarius* and *S. aureus*) produced less marked responses than *P. aeruginosa* and were similar to each other. A co-culture of *P. aeruginosa* and *S. salivarius* produced a structured architecture in the tube, including an area free of plugging at the oxic zone but a large plug with abundant tetrazolium reduction at the microaerobic/anaerobic interface, indicating that together these microbes induced rapid respiration at low oxygen tensions (Supplementary Figure S2b).

Table 1 WinCF quantitative variable responses to pure and co-cultures of CF isolates

	pH	*Plugging (%)*	*Gas (%)*	*Redox (relative % increase)*
Sterile tube	7.5	0	0	NA
Pseudomonas aeruginosa	7.7	99	0	77
Mucoid *Pseudomonas aeruginosa*	7.7	14	0	4
Staphylococcus aureus	7.3	10	0	14
Stenotrophomonas maltophilia	7.7	39	0	30
Streptococcus salivarius	7.1	3	0	27
P. aeruginosa/S. salivarius co-culture	7.4	16	0	19

Abbreviations: CF, cystic fibrosis; NA, not applicable; WinCF, Winogradsky cystic fibrosis.

Patient-specific differences observed with the WinCF system

Sputum samples from seven CF patients and six non-CF individuals were inoculated into the WinCF system and incubated for 48 h. Qualitatively, the CF samples appeared more mucoid and the non-CF samples appeared to contain mostly saliva. The pH in the tubes varied from a mean >7.0 from two CF patients to <5.5, whereas all healthy sputa produced a pH <6.5 in the capillary tubes (Figure 2). Three CF samples produced abundant gas bubbles, while the remaining patients produced little-to-no gas. All non-CF sputa produced at least 7.5% gas in the capillary tubes (Figure 2). Plugging and redox activity were especially variable when inoculated with CF compared with non-CF sputa (Fligner–Killeen test of homogeneity of variance: $P = 0.028$ for plugging, $P = 0.039$ for redox, Supplementary Table S2), where some CF patients had elevated plugging or redox activity and others had little. Non-CF sputa produced low redox activity, except two individuals who had larger amounts of tetrazolium reduction. Plugging, in particular, appears to be a CF-specific response, as all non-CF sputa had little plugging. Overall, the WinCF system demonstrated high variability in the physiology of a CF microbial lung infection. For non-CF sputa, however, the microbial communities had more similar behavior between individuals, except for the redox tubes.

WinCF system fluctuations during a CFPE

Sputum samples ($n = 26$) were collected from a single patient across 198 days through two separate exacerbation events and inoculated into the WinCF system. Forty-five days prior to CFPE1, the system revealed that the microbial community produced a neutral pH in its environment, induced gas bubbles that filled 10% of the media, had moderate respiratory activities and approximately 10% of the tube contained plugging (Figure 3). Large changes in WinCF system variables were observed in the sputum sample taken immediately prior to antibiotic treatment. In the first, more severe exacerbation, the media pH was lowered to ≤5, gas bubbles were significantly elevated to 40% of the media volume, while plugging and respiratory activities were slightly elevated. Responses were similar at the onset of the second exacerbation event, but gas production was 16% lower than that of CFPE1 with a 0.5 unit higher terminal pH. Within 24 h of intravenous antibiotic treatment for CFPE1, drastic changes were observed in the WinCF system. The microbial community raised the media pH to 7.7, gas bubbles were completely eliminated and tube plugging was significantly elevated (Figure 3). The same change in microbial physiology from CFPE1 was seen after the oral treatment of CFPE2, but the effect took days, not hours to be observed (Figure 3). Through the course of intravenous antibiotic therapy, and 4 months of relative disease stability, gas production in the WinCF system never reappeared. The pH of the media fluctuated, but during this time period lowered pH did not correlate to gas production. Redox and plugging activity spiked at days 114–134, with an overall decreasing trend in the amount of respiration. Gas bubbles were not observed again until the onset of the second exacerbation event (Figure 3).

A longitudinal sample that produced bubbles in WinCF tubes (day 195, Figure 3 and Figure 5) was inoculated into another set of capillary tubes and incubated for headspace gas collection and identification of volatile organic compounds (Colman *et al.*, 2001). Of all the significantly elevated headspace gases detected from sputum-inoculated tubes, acetaldehyde and dimethyl disulfide (DMDS) had the greatest fold increase compared with sterile tubes (permutation test, Supplementary Table S3) (Figure 4).

Microbial communities in the WinCF system

The bacterial 16S rRNA gene was amplified and sequenced to assess the taxonomic composition of the microbial communities of the sputum and WinCF capillary tubes in this study. Sputum samples were sequenced in duplicate and the three WinCF capillary tubes for each sample were pooled prior to sequencing. Between CF patients, the microbiomes were very unique, which likely explains the varying responses seen in the WinCF physiochemical variables. In several cases, especially in the absence of intravenous antibiotic treatment, there was a greater abundance of anaerobic bacteria in the capillary samples and larger numbers of *P. aeruginosa* and *Rothia* spp. in the sputum (Figure 5a). During the antibiotic treatment of CFPE1, 8 of the 11 microbial communities profiles in sputa were similar to their corresponding capillary tubes, in that they were both dominated by *P. aeruginosa* (Supplementary Figure S3). Four of the seven cross-sectional WinCF sputum

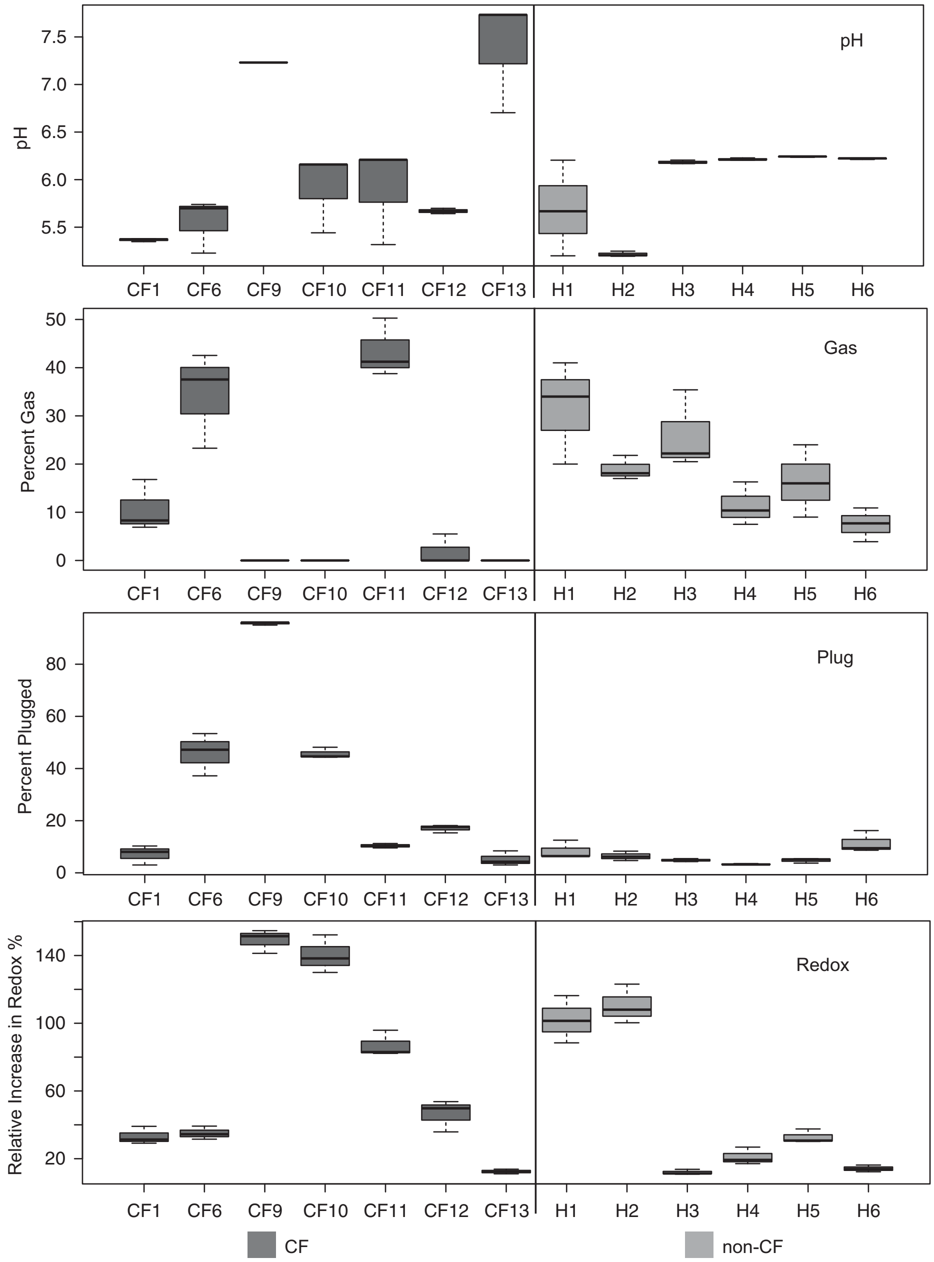

Figure 2 Box plots of the quantitative measures of the triplicate WinCF tubes from the MATLAB output of the capillary tube images. Each tube is measured independently in the MATLAB script, and the distribution of these measures for each sample is shown.

communities were similar to the sputum sample (CF1, 6, 9 and 11), while the other three were very different, although all communities contained abundant *Streptococcus* spp. (Figure 5a, Supplementary Figures S4a and b). Multivariate clustering of the 16S rRNA gene data from WinCF and sputum together did not separate WinCF and sputum for CF samples, but non-CF samples did cluster based on sample type (Supplementary Figure S4c). Partitioning around mediods clustering of the data verified the presence of four clusters: non-CF WinCF communities, non-CF sputum communities and two CF clusters, which were mixed with both sample types (Supplementary Figure S4d). This demonstrated that the WinCF system did not bias the communities to a particular profile for CF samples but may bias non-CF samples to a particular community composition. Instead, for CF samples, the microbial community in the tubes was determined by the sputum sample inoculated, although that community did not always mirror the sputum sample. To further investigate possible biases in the WinCF system, the ratio of the number of reads to individual genera (minimum 5000 total reads) in the sputum were compared with WinCF profiles. The genera with a greater than 10-fold difference in

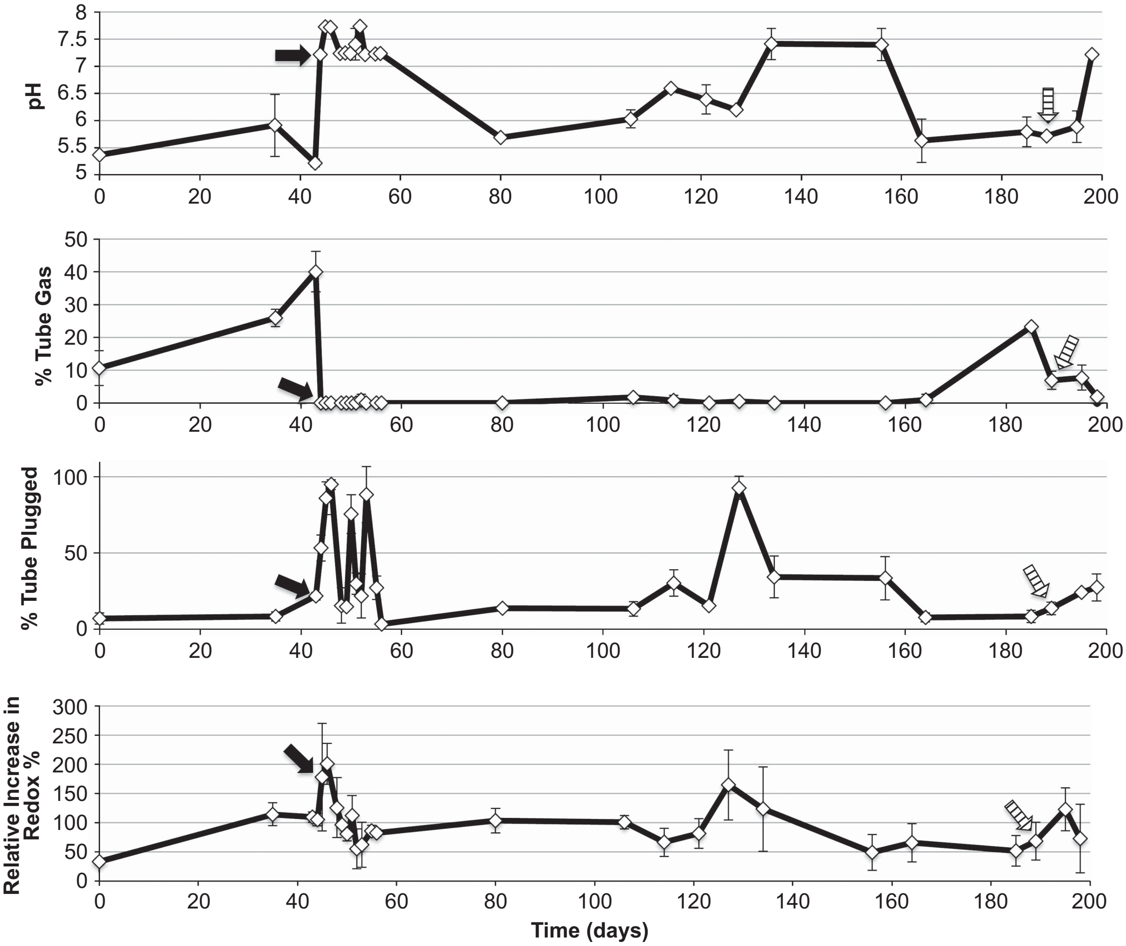

Figure 3 Fluctuations in WinCF model variables through two separate exacerbation events in a single CF patient. Quantitative MATLAB output of the images is plotted through time where each point represents a sputum sample. Arrows in the plot indicate where antibiotics were administered for an exacerbation, the black arrow was intravenous and the striped arrow was oral administration of antibiotics.

abundance were *Rothia*, *Burkholderia*, *Haemophilus* and *Enterobacter*. There was a greater abundance of *Rothia* in the sputum samples compared with the WinCF and a greater abundance of the other three in the WinCF communities (Supplementary Table S4). This may indicate that *Rothia* spp. did not grow well in the WinCF system or that they were not active at the time of sputum sampling. The system may also enrich for the growth of *Burkholderia*, *Enterobacter* and *Haemophilus*. Thus, except for some genera, the WinCF system is not biased for a particular community. WinCF communities did not alter the Shannon diversity of the sputum communities, except for non-CF where the sputum had a significantly higher Shannon index ($P<0.05$, one-tailed Student's *t*-test) (Supplementary Figure S5).

Sequencing the 16S rRNA gene from longitudinal samples through two CF exacerbations revealed major changes in microbial communities in the WinCF capillary tubes. In samples taken just prior to antibiotic treatment, the WinCF and sputum communities contained abundant obligate anaerobes, including *Prevotella*, *Veillonella*, *Gamella* and abundant members of the Lactobacillales, including *Granulicatella* and *Streptococcus* (Figure 5b). Immediately after administration of intravenous antibiotics, the obligate anaerobes were eliminated and the WinCF microbial communities were dominated by *Pseudomonas*. During the first few days of treatment, *Streptococcus*, *Staphylococcus* and *Burkholderia* also grew but then decreased in abundance. After these first few days of treatment, the WinCF communities again became dominated by *Pseudomonas* and remained so, with some *Stenotrophomonas* and *Burkholderia* also observed. After the 14-day treatment course, and during a period of disease stability, the WinCF communities varied with abundant *Streptococcus*, *Granulicatella* and *Pseudomonas*. Upon the second exacerbation event, clinically milder than the first, and treated with oral rather than intravenous antibiotics, the obligate anaerobe-dominated community re-established both 5 days prior and 1 day prior to antibiotic treatment. Oral antibiotic treatment again decreased the abundances of the anaerobic community (*Granulicatella*, *Streptococcus*, *Veillonella*) but did not completely remove them (Figure 5b). During treatment, *Pseudomonas* abundances again increased with the

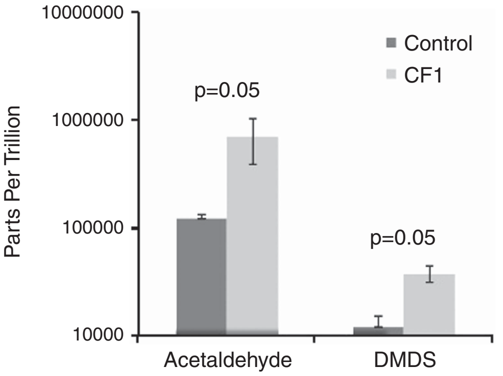

Figure 4 Significantly elevated gasses (permutation test) detected from WinCF capillary tube headspace using gas chromatography and mass spectrometry. Gasses were captured after inoculation with a sputum sample from patient CF1 and incubation at 37 °C in sealed glass containers.

appearance of *S. maltophilia*, but this response took longer to develop, with some *Veillonella* sp. remaining 9 days after oral antibiotic treatment.

A Pearson's correlation test was run on the WinCF bacterial genera abundances and the quantified amount of gas bubbles in the tubes. In both the cross-sectional and exacerbation sample sets, fermentative anaerobes were most correlated to the amount of gas bubbles. Particular genera with the highest correlations included *Prevotella* ($r = 0.73$ cross-sectional and $r = 0.82$ longitudinal), *Veillonella* ($r = 0.84$ longitudinal) and *Fusobacterium* ($r = 0.73$ cross-sectional) (Supplementary Table S5).

Explant lung tissue in the WinCF system

Explant lung tissue from 10 different locations of a CF lung were homogenized and inoculated into the WinCF system. In these samples, there was only significant variation in the amount of plugging or redox activity, while pH was consistently elevated and no gas production was observed. Responses in the WinCF variables were similar regardless of the two-dimensional location of the lung sample, aside from explant samples C, D and E (Supplementary Figure S6). These results were compared with all other samples inoculated into the WinCF system in this study using a Bray–Curtis distance matrix visualized with multidimensional scaling (Figure 6). Seven of the explant lung samples clustered closely in the plot, indicating that they had highly similar features, whereas samples C, D, and E were separate from this group. This may indicate heterogeneity in microbial physiology in this more central section of the lung (Supplementary Figure S6). The explant lung homogenates had a very similar response to that of pure *P. aeruginosa* and a sputum sample dominated by *P. aeruginosa* in the microbiome (CF9, Figure 6). The explant lung samples and *P. aeruginosa* clustered in the plot with clear separation from exacerbation and healthy sputa but were more similar to sputum samples taken at the times of antibiotic treatment. Stable sputum samples were more separated in the multidimensional scaling, indicating that they were more variable than the other sample types, and three cross-sectional sputum samples also clustered with the CFPE samples and non-CF sputum samples.

Discussion

WinCF and the climax and attack model (CAM)

The CAM proposed that there are two functionally different microbial communities in the CF lungs (Conrad *et al.*, 2012). Growth of the Attack Community, consisting of transient viral and microbial populations, is predicted to induce strong immune responses, which create microenvironments that facilitate establishment of a Climax community that is slower growing and inherently resistant to antibiotic therapy (Conrad *et al.*, 2012). Metagenomics studies have shown that the core physiology of the CF microbial community contains both respiratory and fermentative pathways for energy generation (Quinn *et al.*, 2014). In this study, the WinCF system was developed to test the CAM and study the physiology of the CF microbial community. Longitudinal sputum samples through two exacerbation events revealed that fermentative and respiratory physiologies were dynamic through time, and cross-sectional sampling revealed that they were differentially active in different patients (Figures 2 and 3). This result is consistent with the CAM model in this individual and emphasizes the importance of microbial physiology in its dynamics. Furthermore, there is evidence that these two physiologies may differentially affect the lung environment, one that raises the pH and is primarily driven by a respiratory metabolism, and the other, which lowers the environmental pH and is dominated by fermentation. Support for a fermentative metabolism included the decrease in media pH and the production of abundant gas bubbles in the WinCF system. Metabolite analysis of WinCF capillaries inoculated with sputum contained abundant acetaldehyde compared with an uninoculated control (Figure 4). Acetaldehyde is a major fermentation product of pyruvate and alcoholic fermentation and its production from ethanol fermentation has been documented before from oral streptococci (Kurkivuori *et al.*, 2007), which were present in the samples tested in this study (Figure 5).

Sources of the fermentative response

The lower airways are in constant contact with bacteria from the upper airway and the oral cavity due to aspiration, swallowing and other factors (Whiteson *et al.*, 2014). Although healthy lungs are relatively free of clogging mucus, CF airways contain abundant mucus due to disruption of mucus physiology from CFTR mutations and inflammation. The abundant airway mucus in CF creates habitat

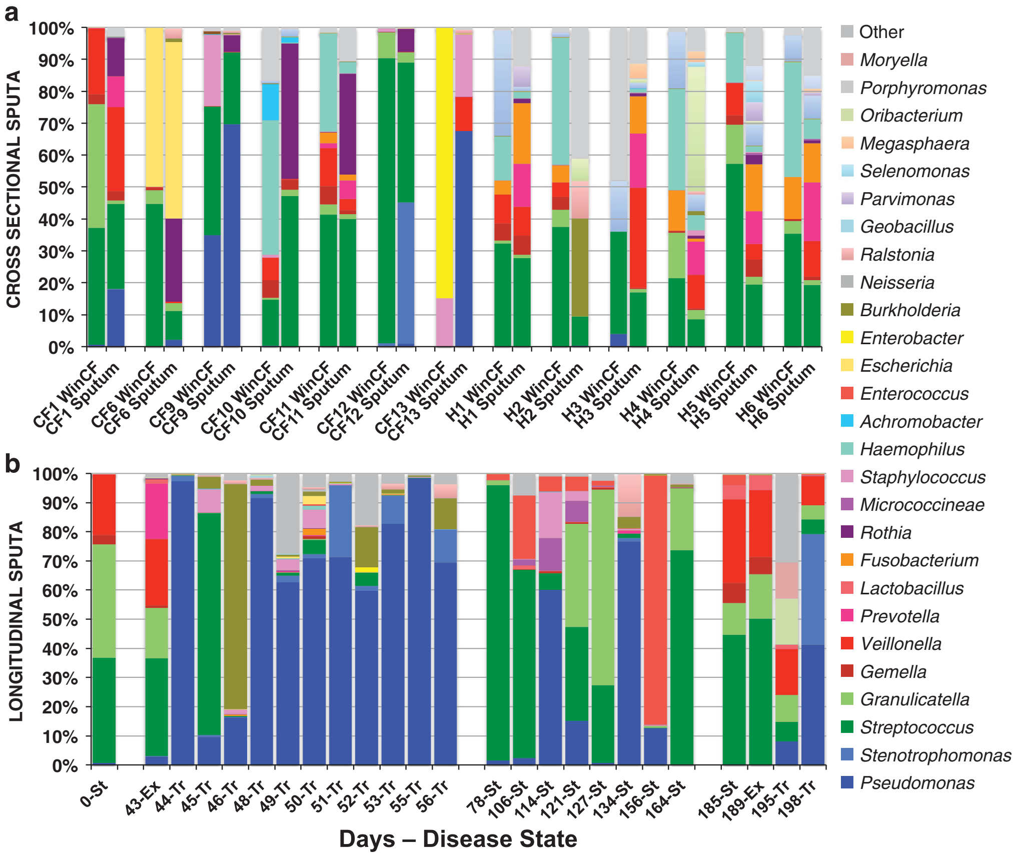

Figure 5 (**a**) Microbial community composition indicated by sequencing of V4 region of 16S rRNA gene from cross-sectional WinCF and sputum samples in both CF and non-CF individuals (genera with at least 5000 total reads from Miseq Illumina sequences analyzed with mothur). Profiles from the same sputum sample are paired in the graph to demonstrate the WinCF community that developed from the same sputum sample. (**b**) 16S rRNA gene microbiome profile of WinCF communities from longitudinal sputum samples through two exacerbations in one patient. The clinically determined disease state is indicated for each sample along with the days since collection of the first sample.

for bacteria from outside the lung to colonize and persist. Accordingly, evidence for oral bacterial colonization, persistence and adaptation to the CF lung has been previously described (Willner *et al.*, 2012; Lim *et al.*, 2013). There is evidence in this study that the fermentative physiology in both non-CF sputum and CF exacerbation samples was contributed by oral anaerobes. Fermentation signals from non-CF sputum likely came from oral anaerobes in saliva, because the absence of abundant lung mucus in a healthy person results in expectoration of large amounts of saliva. However, fermentative signals observed from CF samples were not likely from saliva contamination, because the characteristic gas bubble formation was only present during exacerbation and never at times of stability even though sampling procedures were identical. Oral bacteria have been detected before in the upper airways and sputum of CF patients (Tunney *et al.*, 2008; Worlitzsch *et al.*, 2009; Goddard *et al.*, 2012; Willner *et al.*, 2012), and our results indicate that they colonize lung mucus and may contribute significantly to exacerbations. However, it cannot be known whether these bacteria drive exacerbation onset or whether their growth is favored during the conditions of an exacerbating CF lung. Understanding the causes for the variability in their presence is of particular interest. Some cross-sectional samples contained large fermentative responses with abundant oral microbes, while others had mostly Climax Community members and physiology (Figures 2 and 5). Antibiotic therapy likely contributes significantly to fluctuations in the abundance of these bacteria, because Climax Community members are more antibiotic resistant while Attack Community members are more sensitive to antibiotic treatment (Conrad *et al.*, 2012). The availability of anaerobic niche space is also likely a major factor, which could result from Climax Community growth consuming oxygen or sealing off particular areas of the lung, along with other factors that create a low oxygen mucus environment.

Climax Community and P. aeruginosa

The WinCF system revealed that CF microbial communities have particular properties not seen in non-CF sputum, including plugging of the capillary tube, abundant respiratory activities and

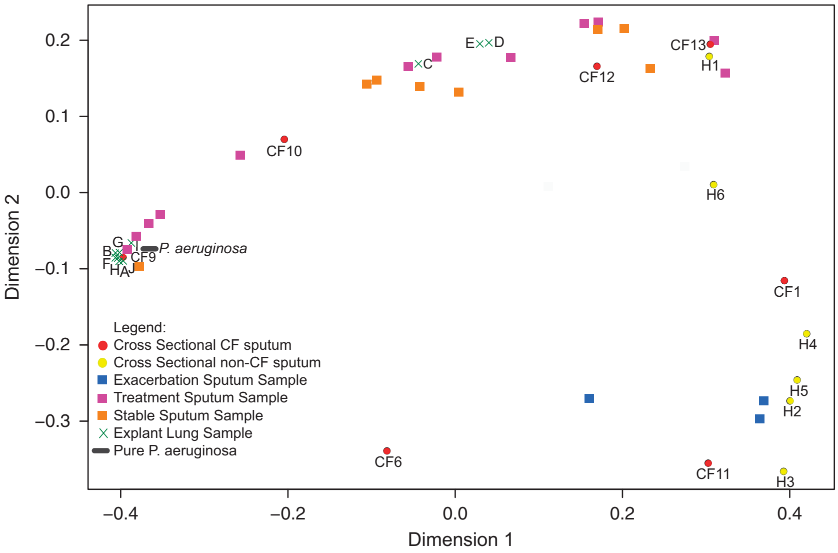

Figure 6 Multidimensional scaling plot of a Bray–Curtis distance matrix comparing output of quantitative WinCF model variables for all samples in this study. Squares are longitudinal sputum samples where blue squares are exacerbation samples, purple squares are during treatment and orange squares are during stability. Circles are cross-sectional sputum samples from different CF (red) and non-CF (yellow) patients. Crosses are various regions of a homogenized explant lung, each spot representing a different location in the lung. The oval is a pure culture of *P. aeruginosa*.

the ability to raise the environmental pH. These characteristics may represent the behavior of classic CF pathogens, such as *P. aeruginosa* and other members of the Climax Community. Accordingly, these responses were particularly characteristic of *P. aeruginosa* and *S. maltophilia* pure cultures, a sputum sample dominated by *P. aeruginosa* (CF9) and an explant lung sample from a *P. aeruginosa*-dominated patient. The behavior of *P. aeruginosa*- and Climax Community-dominated samples matches much of what is recorded in the literature: that the organism grows anaerobically using denitrification in antibiotic-resistant biofilms (Worlitzsch *et al.*, 2002; Palmer *et al.*, 2005; Hogardt and Heesemann, 2010; Schobert and Jahn, 2010). The elevated pH in the media may be due to the production of ammonia from amino-acid breakdown, often observed with *P. aeruginosa* grown in a CF state (Verhoogt *et al.*, 1992; Barth and Pitt, 1996; Thomas *et al.*, 2000; Frimmersdorf *et al.*, 2010), and previously demonstrated to accumulate in WinCF capillary tubes after the growth of CF microbes (Quinn *et al.*, 2014). Thus the *P. aeruginosa* WinCF responses may reflect the true physiology of this microbe and others like it in the CF lung and is in stark contrast to the physiology of the Attack Community during exacerbation events. However, *P. aeruginosa* exhibits large strain differentiation in the CF lungs (Chung *et al.*, 2012; Klockgether *et al.*, 2013), making conceptualizing the organism in the context of CF challenging. Also, while observed as a member of the Climax Community in the patient in this study, it cannot be inferred that *P. aeruginosa* has a limited role in all exacerbations. Of particular interest will be studies on the impact of fermentative anaerobes on *P. aeruginosa* physiology and behavior. For example, the streptococcal fermentation product 2,3-butanediol can induce elevated virulence factor production by *P. aeruginosa*, which may also contribute to the pathology of exacerbations (Venkataraman *et al.*, 2014).

WinCF as a clinical and experimental tool

CF clinical microbiology relies on inherently biased methods that grow CF microbes in a foreign state from which they contribute to disease. The WinCF system represents an effective method of providing clinically relevant information about a CF patient's infection that is rapid and extremely cost effective. It is an excellent experimental tool that can reveal the behavior of CF microbes in an environment more similar to the CF lung. For example, DMDS was detected in headspace gas from WinCF capillary tubes and breath gas samples from CF patients (Kamboures *et al.*, 2005; Barker *et al.*, 2006). DMDS is a major by-product of methionine degradation (Segal and Starkey, 1968), and methionine is a major component of the mucin glycopeptide. The WinCF system may also be effective for the study of mucin degradation by bacteria and the by-products produced in the sulfur-rich environment of a mucus-filled lung. The WinCF system does not completely mimic a CF lung bronchiole, because the fundamental role of host cells is mostly removed, and the media used is merely a mimic of CF sputum based on measured chemistry. However, the system allows for an amplification and visual interpretation of the behavior of the microbes and allows for a better understanding of how bacteria may influence the CF lung environment.

The WinCF microbial communities did not always mimic that of the sputum from which they were inoculated; however, during antibiotic treatment the profiles were similar. Partitioning around mediods clustering of the 16S rRNA gene sequence data revealed that there were four strong clusters of cross-sectional microbiome communities. Two of these clusters were comprised of capillary tube non-CF and sputum non-CF communities, but the other two clusters were a mix of capillary tube CF and sputum CF communities (Supplementary Figures S4c and d). This indicated that while there may be some bias for healthy sputum using the WinCF system, as the capillary tubes tended to enrich for similar microbial communities, the system was not biased to a particular community for CF samples. However, there was a notable bias for the growth of *Rothia* spp. Abundant reads of the 16S rRNA gene data were assigned to *Rothia* from the sputum samples, but these were virtually absent from the WinCF communities after incubation. This indicated that while *Rothia* was inoculated into the system, it did not grow, either due to its fastidiousness or lack of appropriate conditions. Another possibility is that there is bias in the WinCF system against non-motile bacteria. *Rothia*, a non-motile Gram-positive, did not grow well in the capillary tubes, and the mucoid isolate of *P. aeruginosa*, also without a flagellum, produced $7\times$ less biofilm than a non-mucoid and motile strain. Alternatively, *Rothia* may have been dormant in the sputum samples at the time of collection, its DNA in the sputum amplified by PCR from either dead cells or extracellular sources. The WinCF system may enrich for actively growing microbes, and future work is needed to investigate discrepancies between active microbes in a sputum sample and microbiome profiles by 16S rRNA gene amplification, which can be susceptible to sequencing ‘dead’ DNA (Wolffs *et al.*, 2005; Young *et al.*, 2007; Rogers *et al.*, 2010).

Conclusion

There has been much debate about whether the CF microbial community changes during exacerbation development. Some studies find little change (Fodor *et al.*, 2012; Deschaght *et al.*, 2013; Price *et al.*, 2013), while others are finding fluctuations in anaerobic microbial abundance (Carmody *et al.*, 2013; Deschaght *et al.*, 2013; Twomey *et al.*, 2013; Zemanick *et al.*, 2013). The WinCF system demonstrated that obligate anaerobes are abundant prior to exacerbation and actively fermenting. Antibiotic treatment quickly eliminated these bacteria, indicating that the CF microbial community was dynamic through exacerbation events in this patient. Similar signatures of microbial fermentation have been described in other studies (Whiteson *et al.*, 2014), including in exacerbation samples (Twomey *et al.*, 2013), and the anaerobe *Gamella* was the most discriminate genus between exacerbation and stable samples in a previous microbiome study (Carmody *et al.*, 2013). In light of these results, we propose that the CAM, originally described by Conrad *et al.* (2012), should be focused on microbial physiology. The Attack Community may be fermentative, and the production of acidic and gaseous products from this community may contribute significantly to exacerbation pathogenesis. The Climax Community, on the other hand, may be primarily respiratory, slow growing and highly antibiotic resistant. Earlier and effective therapies against anaerobes with specific antibiotics or chemical treatments, such as hyperbaric oxygen, could change the treatment of CFPE and improve the quality and duration of life of CF patients.

Conflict of Interest

The authors declare no conflict of interest.

Acknowledgements

This work was supported by the National Institute of Health through grant R01 GM095384-01 awarded to Forest Rohwer. The project was partially supported by the National Institutes of Health, grant UL1RR031980 for years 1 and 2 of CTSA funding and/or UL1TR000100 during year 3 and beyond of CTSA funding. Funding was also provided by the Canadian Institute for Advanced Research and by the Cystic Fibrosis Research Inc. Elizabeth Nash memorial fellowship awarded to Robert Quinn. We also thank Mark Hatay and Amadeo Candido for their contributions to the imaging set up and mucus_analyzer script development.

References

Barker M, Hengst M, Schmid J, Buers H-J, Mittermaier B, Klemp D *et al.* (2006). Volatile organic compounds in the exhaled breath of young patients with cystic fibrosis. *Eur Respir J* **27**: 929–936.

Barth AL, Pitt TL. (1996). The high amino-acid content of sputum from cystic fibrosis patients promotes growth of auxotrophic *Pseudomonas aeruginosa*. *J Med Microbiol* **45**: 110–119.

Bjarnsholt T, Jensen PØ, Fiandaca MJ, Pedersen J, Hansen CR, Andersen CB *et al.* (2009). *Pseudomonas aeruginosa* biofilms in the respiratory tract of cystic fibrosis patients. *Pediatr Pulmonol* **44**: 547–558.

Burns JL, Rolain J-M. (2014). Culture-based diagnostic microbiology in cystic fibrosis: can we simplify the complexity? *J Cyst Fibros* **13**: 1–9.

Caporaso JG, Lauber CL, Walters WA, Berg-Lyons D, Lozupone CA, Turnbaugh PJ *et al.* (2011). Global patterns of 16S rRNA diversity at a depth of millions of sequences per sample. *Proc Natl Acad Sci USA* **108 Suppl**: 4516–4522.

Carmody LA, Zhao J, Schloss PD, Petrosino JF, Murray S, Young VB *et al.* (2013). Changes in cystic fibrosis

airway microbiota at pulmonary exacerbation. *Ann Am Thorac Soc* **10**: 179–187.

Chung JCS, Becq J, Fraser L, Schulz-Trieglaff O, Bond NJ, Foweraker J *et al.* (2012). Genomic variation among contemporary *Pseudomonas aeruginosa* isolates from chronically infected cystic fibrosis patients. *J Bacteriol* **194**: 4857–4866.

Cole JR, Chai B, Farris RJ, Wang Q, Kulam SA, McGarrell DM *et al.* (2005). The Ribosomal Database Project (RDP-II): sequences and tools for high-throughput rRNA analysis. *Nucleic Acids Res* **33**: D294–D296.

Colman JJ, Swanson AL, Meinardi S, Sive BC, Blake DR, Rowland FS. (2001). Description of the analysis of a wide range of volatile organic compounds in whole air samples collected during PEM-tropics A and B. *Anal Chem* **73**: 3723–3731.

Conrad D, Haynes M, Salamon P, Rainey PB, Youle M, Rohwer F. (2012). Cystic fibrosis therapy: a community ecology perspective. *Am J Respir Cell Mol Biol* **48**: 150–156.

De Boer K, Vandemheen KL, Tullis E, Doucette S, Fergusson D, Freitag A *et al.* (2011). Exacerbation frequency and clinical outcomes in adult patients with cystic fibrosis. *Thorax* **66**: 680–685.

Delhaes L, Monchy S, Fréalle E, Hubans C, Salleron J, Leroy S *et al.* (2012). The airway microbiota in cystic fibrosis: a complex fungal and bacterial community–implications for therapeutic management. *PLoS One* **7**: e36313.

Deschaght P, Schelstraete P, Van Simaey L, Vanderkercken M, Raman A, Mahieu L *et al.* (2013). Is the improvement of CF patients, hospitalized for pulmonary exacerbation, correlated to a decrease in bacterial load? *PLoS One* **8**: e79010.

Duan K, Dammel C, Stein J, Rabin H, Surette MG. (2003). Modulation of *Pseudomonas aeruginosa* gene expression by host microflora through interspecies communication. *Mol Microbiol* **50**: 1477–1491.

Fenchel T, Finlay B. (2008). Oxygen and the spatial structure of microbial communities. *Biol Rev Camb Philos Soc* **83**: 553–569.

Fodor Aa, Klem ER, Gilpin DF, Elborn JS, Boucher RC, Tunney MM *et al.* (2012). The adult cystic fibrosis airway microbiota is stable over time and infection type, and highly resilient to antibiotic treatment of exacerbations. *PLoS One* **7**: e45001.

Frimmersdorf E, Horatzek S, Pelnikevich A, Wiehlmann L, Schomburg D. (2010). How *Pseudomonas aeruginosa* adapts to various environments: a metabolomic approach. *Environ Microbiol* **12**: 1734–1747.

Fuchs HJ, Borowitz DS, Christiansen DH, Morris EM, Nash ML, Ramsey BW *et al.* (1994). Effect of aerosolized recombinant human DNase on exacerbations of respiratory symptoms and on pulmonary function in patients with cystic fibrosis. The Pulmozyme Study Group. *N Engl J Med* **331**: 637–642.

Fung C, Naughton S, Turnbull L, Tingpej P, Rose B, Arthur J *et al.* (2010). Gene expression of *Pseudomonas aeruginosa* in a mucin-containing synthetic growth medium mimicking cystic fibrosis lung sputum. *J Med Microbiol* **59**: 1089–1100.

Ghio AJ, Roggli VL, Soukup JM, Richards JH, Randell SH, Muhlebach MS. (2012). Iron accumulates in the lavage and explanted lungs of cystic fibrosis patients. *J Cyst Fibros* **12**: 390–398.

Goddard AF, Staudinger BJ, Dowd SE, Joshi-Datar A, Wolcott RD, Aitken ML *et al.* (2012). Direct sampling of cystic fibrosis lungs indicates that DNA-based analyses of upper-airway specimens can misrepresent lung microbiota. *Proc Natl Acad Sci USA* **109**: 13769–13774.

Grasemann H, Ioannidis I, Tomkiewicz RP, de Groot H, Rubin BK, Ratjen F. (1998). Nitric oxide metabolites in cystic fibrosis lung disease. *Arch Dis Child* **78**: 49–53.

Haas AF, Gregg AK, Smith JE, Abieri ML, Hatay M, Rohwer F. (2013). Visualization of oxygen distribution patterns caused by coral and algae. *PeerJ* **1**: e106.

Hare NJ, Soe CZ, Rose B, Harbour C, Codd R, Manos J *et al.* (2012). Proteomics of *Pseudomonas aeruginosa* Australian epidemic strain 1 (AES-1) cultured under conditions mimicking the cystic fibrosis lung reveals increased iron acquisition via the siderophore pyochelin. *J Proteome Res* **11**: 776–795.

Harrison F. (2007). Microbial ecology of the cystic fibrosis lung. *Microbiology* **153**: 917–923.

Hauser AR, Jain M, Bar-Meir M, McColley Sa. (2011). Clinical significance of microbial infection and adaptation in cystic fibrosis. *Clin Microbiol Rev* **24**: 29–70.

Hill D, Rose B, Pajkos A, Robinson M, Bye P, Bell S *et al.* (2005). Antibiotic susceptabilities of *Pseudomonas aeruginosa* isolates derived from patients with cystic fibrosis under aerobic, anaerobic, and biofilm conditions. *J Clin Microbiol* **43**: 5085–5090.

Hogardt M, Heesemann J. (2010). Adaptation of *Pseudomonas aeruginosa* during persistence in the cystic fibrosis lung. *Int J Med Microbiol* **300**: 557–562.

Hunter RC, Klepac-Ceraj V, Lorenzi MM, Grotzinger H, Martin TR, Newman DK. (2012). Phenazine content in the cystic fibrosis respiratory tract negatively correlates with lung function and microbial complexity. *Am J Respir Cell Mol Biol* **47**: 738–745.

Jones KL, Hegab AH, Hillman BC, Simpson KL, Jinkins PA, Grisham MB *et al.* (2000). Elevation of nitrotyrosine and nitrate concentrations in cystic fibrosis sputum. *Pediatr Pulmonol* **30**: 79–85.

Kamboures MA, Blake DR, Cooper DM, Newcomb RL, Barker M, Larson JK *et al.* (2005). Breath sulfides and pulmonary function in cystic fibrosis. *Proc Natl Acad Sci USA* **102**: 15762–15767.

King P, Citron DM, Griffith DC, Lomovskaya O, Dudley MN. (2010). Effect of oxygen limitation on the in vitro activity of levofloxacin and other antibiotics administered by the aerosol route against *Pseudomonas aeruginosa* from cystic fibrosis patients. *Diagn Microbiol Infect Dis* **66**: 181–186.

Klockgether J, Miethke N, Kubesch P, Bohn Y-S, Brockhausen I, Cramer N *et al.* (2013). Intraclonal diversity of the *Pseudomonas aeruginosa* cystic fibrosis airway isolates TBCF10839 and TBCF121838: distinct signatures of transcriptome, proteome, metabolome, adherence and pathogenicity despite an almost identical genome sequence. *Environ Microbiol* **15**: 191–210.

Kozich JJ, Westcott SL, Baxter NT, Highlander SK, Schloss PD. (2013). Development of a dual-index sequencing strategy and curation pipeline for analyzing amplicon sequence data on the MiSeq Illumina sequencing platform. *Appl Environ Microbiol* **79**: 5112–5120.

Kurkivuori J, Salaspuro V, Kaihovaara P, Kari K, Rautemaa R, Grönroos L *et al.* (2007). Acetaldehyde production from ethanol by oral streptococci. *Oral Oncol* **43**: 181–186.
Lim YW, Evangelista JS, Schmieder R, Bailey B, Haynes M, Furlan M *et al.* (2013). Clinical insights from metagenomic analysis of cystic fibrosis sputum. *J Clin Microbiol* **52**: 425–457.
Lim YW, Schmieder R, Haynes M, Furlan M, Matthews TD, Whiteson K *et al.* (2013). Mechanistic model of Rothia mucilaginosa adaptation toward persistence in the CF lung, based on a genome reconstructed from metagenomic data. *PLoS One* **8**: e64285.
Lim YW, Schmieder R, Haynes M, Willner D, Furlan M, Youle M *et al.* (2012). Metagenomics and metatranscriptomics: Windows on CF-associated viral and microbial communities. *J Cyst Fibros* **12**: 164–154.
Lipuma JJ. (2010). The changing microbial epidemiology in cystic fibrosis. *Clin Microbiol Rev* **23**: 299–323.
Mitchell G, Séguin DL, Asselin A-E, Déziel E, Cantin AM, Frost EH *et al.* (2010). Staphylococcus aureus sigma B-dependent emergence of small-colony variants and biofilm production following exposure to *Pseudomonas aeruginosa* 4-hydroxy-2-heptylquinoline-N-oxide. *BMC Microbiol* **10**: 33.
Morris RL, Schmidt TM. (2013). Shallow breathing: bacterial life at low O(2). *Nat Rev Microbiol* **11**: 205–212.
Palmer KL, Aye LM, Whiteley M. (2007). Nutritional cues control *Pseudomonas aeruginosa* multicellular behavior in cystic fibrosis sputum. *J Bacteriol* **189**: 8079–8087.
Palmer KL, Mashburn LM, Singh PK, Whiteley M. (2005). Cystic fibrosis sputum supports growth and cues key aspects of *Pseudomonas aeruginosa* physiology. *J Bacteriol* **187**: 5267–5277.
Pezzulo AA, Tang XX, Hoegger MJ, Alaiwa MHA, Ramachandran S, Moninger TO *et al.* (2012). Reduced airway surface pH impairs bacterial killing in the porcine cystic fibrosis lung. *Nature* **487**: 109–113.
Price KE, Hampton TH, Gifford AH, Dolben EL, Hogan DA, Morrison HG *et al.* (2013). Unique microbial communities persist in individual cystic fibrosis patients throughout a clinical exacerbation. *Microbiome* **1**: 27.
Quast C, Pruesse E, Yilmaz P, Gerken J, Schweer T, Yarza P *et al.* (2013). The SILVA ribosomal RNA gene database project: improved data processing and web-based tools. *Nucleic Acids Res* **41**: D590–D596.
Quinn RA, Lim YW, Maughan H, Conrad D, Rohwer F, Whiteson KL. (2014). Biogeochemical forces shape the composition and physiology of polymicrobial communities in the cystic fibrosis lung. *MBio* **5**: e00956–13.
Rabin HR, Surette MG. (2012). The cystic fibrosis airway microbiome. *Curr Opin Pulm Med* **18**: 622–627.
Reid DW, Lam QT, Schneider H, Walters EH. (2004). Airway iron and iron-regulatory cytokines in cystic fibrosis. *Eur Respir J* **24**: 286–291.
Rogers GB, Marsh P, Stressmann aF, Allen CE, Daniels TVW, Carroll MP *et al.* (2010). The exclusion of dead bacterial cells is essential for accurate molecular analysis of clinical samples. *Clin Microbiol Infect* **16**: 1656–1658.
Schmieder R, Edwards R. (2011). Quality control and preprocessing of metagenomic datasets. *Bioinformatics* **27**: 863–864.
Schobert M, Jahn D. (2010). Anaerobic physiology of *Pseudomonas aeruginosa* in the cystic fibrosis lung. *Int J Med Microbiol* **300**: 549–556.
Segal W, Starkey RL. (1968). Methionine and identity of the microbial decomposition of methionine and identity of the resulting sulfur products. *J Bacteriol* **98**: 908–913.
Shin H-W, Umber BJ, Meinardi S, Leu S-Y, Zaldivar F, Blake DR *et al.* (2009). Acetaldehyde and hexanaldehyde from cultured white cells. *J Transl Med* **7**: 31.
Sibley CD, Parkins MD, Rabin HR, Duan K, Norgaard JC, Surette MG. (2008). A polymicrobial perspective of pulmonary infections exposes an enigmatic pathogen in cystic fibrosis patients. *PNAS* **105**: 15070–15075.
Sibley CD, Surette MG. (2011). The polymicrobial nature of airway infections in cystic fibrosis: Cangene Gold Medal Lecture. *Can J Microbiol* **57**: 69–77.
Stenbit AE, Flume Pa. (2011). Pulmonary exacerbations in cystic fibrosis. *Curr Opin Pulm Med* **17**: 442–447.
Stites SW, Walters B, O'Brien-Ladner A, Bailey K, Wesselius L. (1998). Increased iron and ferritin content of sputum from patients with cystic fibrosis or chronic bronchitis. *Chest* **114**: 814.
Stressmann Fa, Rogers GB, Marsh P, Lilley AK, Daniels TWV, Carroll MP *et al.* (2011). Does bacterial density in cystic fibrosis sputum increase prior to pulmonary exacerbation? *J Cyst Fibros* **10**: 357–365.
Tate S, MacGregor G, Davis M, Innes JA, Greening AP. (2002). Airways in cystic fibrosis are acidified: detection by exhaled breath condensate. *Thorax* **57**: 926–929.
Thomas SR, Ray A, Hodson ME, Pitt TL. (2000). Increased sputum amino acid concentrations and auxotrophy of *Pseudomonas aeruginosa* in severe cystic fibrosis lung disease. *Thorax* **55**: 795–797.
Tunney MM, Field TR, Moriarty TF, Patrick S, Doering G, Muhlebach MS *et al.* (2008). Detection of anaerobic bacteria in high numbers in sputum from patients with cystic fibrosis. *Am J Respir Crit Care Med* **177**: 995–1001.
Twomey KB, Alston M, An S-Q, O'Connell OJ, McCarthy Y, Swarbreck D *et al.* (2013). Microbiota and metabolite profiling reveal specific alterations in bacterial community structure and environment in the cystic fibrosis airway during exacerbation. *PLoS One* **8**: e82432.
Umber BJ, Shin H-W, Meinardi S, Leu S-Y, Zaldivar F, Cooper DM *et al.* (2013). Gas signatures from *Escherichia coli* and *Escherichia coli*-inoculated human whole blood. *Clin Transl Med* **2**: 13.
Venkataraman A, Rosenbaum MA, Werner JJ, Winans SC, Angenent LT. (2014). Metabolite transfer with the fermentation product 2,3-butanediol enhances virulence by *Pseudomonas aeruginosa*. *ISME J* **8**: 1210–1220.
Verhoogt HJ, Smit H, Abee T, Gamper M, Driessen aJ, Haas D *et al.* (1992). arcD, the first gene of the arc operon for anaerobic arginine catabolism in *Pseudomonas aeruginosa*, encodes an arginine-ornithine exchanger. *J Bacteriol* **174**: 1568–1573.
Whiteson KL, Bailey B, Bergkessel M, Conrad D, Delhaes L, Felts B *et al.* (2014). The upper respiratory

tract as a microbial source for pulmonary infections in cystic fibrosis. Parallels from island biogeography. *Am J Respir Crit Care Med* **189**: 1309–1315.
Whiteson KL, Meinardi S, Lim YW, Schmieder R, Maughan H, Quinn R *et al.* (2014). Breath gas metabolites and bacterial metagenomes from cystic fibrosis airways indicate active pH neutral 2,3-butanedione fermentation. *ISME J* **8**: 1247–1258.
Willner D, Furlan M, Haynes M, Schmieder R, Angly FE, Silva J *et al.* (2009). Metagenomic analysis of respiratory tract DNA viral communities in cystic fibrosis and non-cystic fibrosis individuals. *PLoS One* **4**: e7370.
Willner D, Haynes MR, Furlan M, Schmieder R, Lim YW, Rainey PB *et al.* (2012). Spatial distribution of microbial communities in the cystic fibrosis lung. *ISME J* **6**: 471–474.
Winogradsky SN. (1897). O roli mikrobov v krugovorote zhizni (On the role of microbes in the cycle of life). *Arkhiv Biol Nauk* **7**: 1–27.
Wolffs P, Norling B, Rådström P. (2005). Risk assessment of false-positive quantitative real-time PCR results in food, due to detection of DNA originating from dead cells. *J Microbiol Methods* **60**: 315–323.
Worlitzsch D, Rintelen C, Böhm K, Wollschläger B, Merkel N, Borneff-Lipp M *et al.* (2009). Antibiotic-resistant obligate anaerobes during exacerbations of cystic fibrosis patients. *Clin Microbiol Infect* **15**: 454–460.
Worlitzsch D, Tarran R, Ulrich M, Schwab U, Cekici A, Meyer KC *et al.* (2002). Effects of reduced mucus oxygen concentration in airway Pseudomonas infections of cystic fibrosis patients. *J Clin Invest* **109**: 317–325.
Yang L, Jelsbak L, Molin S. (2011). Microbial ecology and adaptation in cystic fibrosis airways. *Environ Microbiol* **13**: 1682–1689.
Yao W, Byrne RH. (2001). Spectrophotometric determination of freshwater pH using bromocresol purple and phenol red. *Environ Sci Technol* **35**: 1197–1201.
Young G, Turner S, Davies JK, Sundqvist G, Figdor D. (2007). Bacterial DNA persists for extended periods after cell death. *J Endod* **33**: 1417–1420.
Zemanick ET, Harris JK, Wagner BD, Robertson CE, Sagel SD, Stevens MJ *et al.* (2013). Inflammation and airway microbiota during cystic fibrosis pulmonary exacerbations. *PLoS One* **8**: e62917.
Zhao J, Schloss PD, Kalikin LM, Carmody La, Foster BK, Petrosino JF *et al.* (2012). Decade-long bacterial community dynamics in cystic fibrosis airways. *Proc Natl Acad Sci USA* **109**: 5809–5814.

Supplementary Information accompanies this paper on The ISME Journal website (http://www.nature.com/ismej)
