# Supplementary material for: Metabolomics of pulmonary exacerbations reveals the personalized nature of cystic fibrosis disease
Source: PeerJ. 2016 Aug 11;4:e2174. doi: 10.7717/peerj.2174 (PMC4991883; doi:10.7717/peerj.2174)
Supplement: Supplemental Information 3 — Supplementary results describing technical variability and batch effects [file peerj-04-2174-s009.docx]

**Supplementary Results**

*Assessment of Method Variability and Batch Collection Effects.* To assess the degree of reproducibility of the method used in this study on a single sample, four sputum samples were collected and analyzed using the same extraction, LC-MS/MS, and data analysis pipeline. Three aliquots of one sample were taken to assess variability within a sample and three more aliquots were subsequently collected on 3 successive days to assess variability day-to-day on the sample metabolome. Visualization of the metabolome relationships from these samples using nMDS of a Bray-Curtis distance matrix demonstrated that our method was highly reproducible within a single batch and a single sample (Fig. S1a). All replicate samples from a single patient clustered tightly together and away form the other patient samples, verifying the within batch reproducibility of the metabolomics analysis procedures used in this study.

After feature finding on the entire data set (both the 2012 and 2014 collections) using Bruker® ProfileAnalysis software, the similarity between the samples was calculated using the Bray-Curtis distance and the first two principle components visualized with principle component analysis (PCoA) (Fig. S1b). This demonstrated that there were significant batch effects between the two collections as there was strong clustering by each individual collection. Thus, multivariate clustering and statistical comparisons were only done within batch.

All mass spectrometry and extraction procedures were identical for samples in this study, thus, we could identify shared compounds across the batches of data. To do this an advanced retention time alignment and feature finding algorithm based on the XCMS CentWave algorithm (see supplementary methods) was applied to identify common peaks across both the 2012 and 2014 multiple patient collections and the 4-year longitudinal collection from CF1. This merged molecular feature table was then used for biomarker discovery across different clinical states in the data set and the longitudinal CF1 collection. A total of 58,311 features were found across all batches and 26,373 batch specific features were removed. Visualizing this merged data set revealed that most of the batch effect signals were within the first 60s and last 120s of the run (which are both designed as column wash stages), indicating they were likely solvent and instrument contaminants varying between the two runs (Fig. S1c).
